# Design, Synthesis and Biological Evaluation of Stilbene Derivatives as Novel Inhibitors of Protein Tyrosine Phosphatase 1B

**DOI:** 10.3390/molecules21121722

**Published:** 2016-12-16

**Authors:** Haibing He, Yinghua Ge, Hong Dai, Song Cui, Fei Ye, Jia Jin, Yujun Shi

**Affiliations:** 1 College of Chemistry and Chemical Engineering, Nantong University, Nantong 226019, China; hehaibing@ntu.edu.cn (H.H.); cuisong123@163.com (S.C.); yefei@zstu.edu.cn (F.Y.); 2 College of Life Sciences, Zhejiang Sci-Tech University, Hangzhou 310018, China; geyinghua1992@163.com

**Keywords:** protein tyrosine phosphatase 1B (PTP1B), lithocholic acid, stilbene, inhibitor

## Abstract

By imitating the scaffold of lithocholic acid (LCA), a natural steroidal compound displaying Protein Tyrosine Phosphatase 1B (PTP1B) inhibitory activity, a series of stilbene derivatives containing phenyl-substituted isoxazoles were designed and synthesized. The structures of the title compounds were confirmed by ^1^H-NMR, ^13^C-NMR and HRMS. Activities of the title compounds were evaluated on PTP1B and the homologous enzyme TCPTP by using a colorimetric assay. Most of the target compounds had good activities against PTP1B. Among them, compound **29** (IC_50_ = 0.91 ± 0.33 μM), characterized by a 5-(2,3-dichlorophenyl) isoxazole moiety, exhibited an activity about 14-fold higher than the lead compound LCA and a 4.2-fold selectivity over TCPTP. Compound **29** was identified as a competitive inhibitor of PTP1B with a *K*_i_ value of 0.78 μM in enzyme kinetic studies.

## 1. Introduction

Protein tyrosine phosphatases (PTPs) are a superfamily of enzymes involved in crucial cellular signalling mechanisms by controlling the phosphorylation levels of specific tyrosine residues in peptides and proteins which regulate many cellular functions, such as cell proliferation, survival, metabolism, adhesion and migration. Among the PTPs so far identified, the cytosolic protein tyrosine phosphatase 1B (PTP1B) was the first phosphatase to be isolated and is therefore the most widely studied [1,2,3,4]. A large body of experimental data acquired from in vivo studies on humans and PTP1B-knockout mice has identified PTP1B as a major negative regulator of both insulin and leptin signals. Consequently, inhibiting of PTP1B was considered to be a potential therapeutics for treating Type 2 diabetes and obesity by enhance insulin sensitivity and resistance to obesity [5,6,7,8]. Moreover, it is worth noting that PTPs share a high degree of structural conservation in the active site, especially for T-cell protein tyrosine phosphatase (TCPTP), which has a sequence identity of about 74% in the catalytic domains with PTP1B [9]. There is always a challenge for the potent PTP1B inhibitors to keep good selectivity over TCPTP in the same time [10].

**Figure 1:**
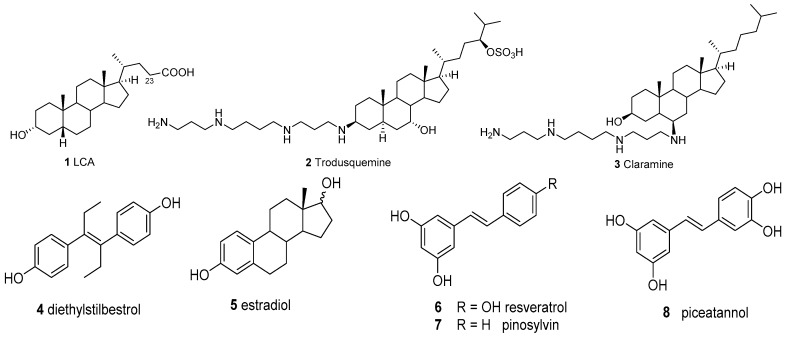
Steroids **1**~**3** reported as PTP1B inhibitors and stilbene derivatives **4**~**8**.

In the past decades, several steroids and their derivatives were found to possess moderate to good PTP1B inhibitory activities, such as lithocholic acid (LCA, **1**, Figure 1) [11], claramine (**3**) [12] and trodusquemine (**2**) [13], the first PTP1B inhibitor for the treatment of diabetes in clinical trials. However, risk of the hormonal effect of steroids and their derivatives always cannot be neglected. Years ago, diethylstilbestrol (**4**) was developed as a mimic and alternative drug for estradiol (**5**) [14], which indicated the fact that the stilbene moiety could be an ideal replacement of the steroid skeleton in drug design when use the “scaffold hopping” method [15]. Additionally, stilbene is a common moiety found in many natural products endowed with antibacterial, antioxidative, antiproliferative or antidiabetic activities, such as resveratrol (**6**), pinosylvin (**7**) and piceatannol (**8**) [16,17,18,19].

**Figure 2:**
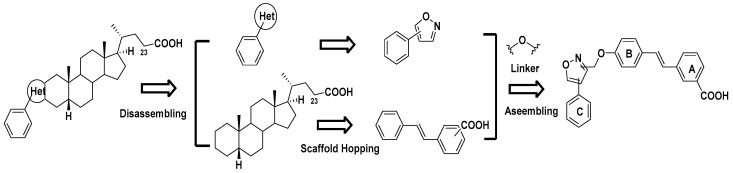
The design of the title compounds.

In a previous report we disclosed that the introduction of phenyl-substituted heterocyclic rings, such as pyrazole, oxazole, isoxazole and thiazoles, into the ring A on LCA would improve the inhibitory activity against PTP1B. On the other hand, it has also been verified that the 23-COOH of LCA was irreplaceable for maintaining the activities against PTP1B [11]. Based on the views above, we tentatively designed a class of stilbene derivatives, as shown in Figure 2, which was characterized by a phenyl-substituted isoxazole linked to the stilbene scaffold by an ether bond. In order to search for the optimized phenyl isoxazole moiety, at the very beginning, we designed and synthesized four mimics (compounds **15**, **16**, **17** and **18**), containing 3-phenyl-4-isoxazolyl-, 5-methyl-3-phenyl-4-isoxazolyl-, 5-phenyl-4-isoxazolyl and 5-phenyl-3-isoxazolyl groups, respectively, and evaluated their inhibitory effects against PTP1B. As a result, compound **18** was found to have the highest activity to PTP1B (IC_50_ = 6.33 ± 1.02 μM). Then a series of its derivatives **19**~**30** with different groups on the phenyl ring C was synthesized and evaluated.

## 2. Results and Discussion

### 2.1. Chemistry

**Scheme 1:**
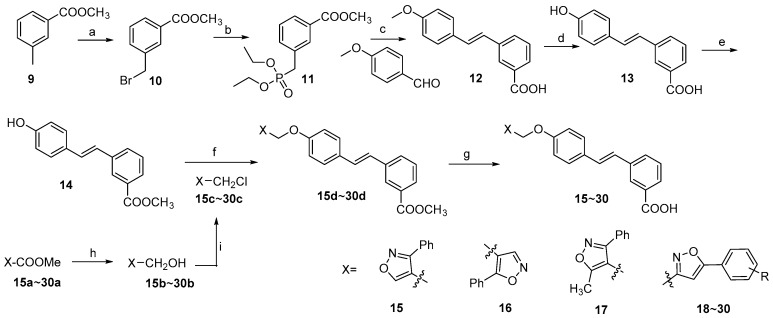
Synthesis of compounds **10**~**30**. *Reagents and conditions:* (**a**) CCl_4_, NBS, reflux, 6 h; (**b**) P(OEt)_3_, 160 °C; (**c**) *t*-BuOK, THF, 0 °C~r.t., overnight; (**d**) AlCl_3_, Et_3_N, 60 °C~90 °C, 18 h; (**e**) MeI, K_2_CO_3_, acetone, 60 °C, 15 h; (**f**) Cs_2_CO_3_, DMSO, 65 °C, 8 h; (**g**) KOH, H_2_O, 65 °C, 6 h; (**h**) LiAlH_4_, 0 °C~r.t., 4 h; (**i**) SOCl_2_, DCM, 45 °C, 8 h.

As shown in Scheme 1, target compounds **15**~**30** were all prepared by condensation of the stilbene intermediate **14** and the corresponding chloromethyl isoxazole **15c**~**30c** in DMSO with Cs_2_CO_3_ as the acid-binding agent, followed by hydrolysis in KOH solution and acidification with hydrochloric acid. Starting from 3-methylbenzoate (**9**), the key intermediate **14** was synthesized in five steps including bromination [20], Arbuzov reaction, Wittig-Horner reaction [21], demethylation [22] and selective methylation. At the beginning, by using PPh_3_, a Wittig reaction was employed to synthesize the compound **12**, giving a mixture of *cis*- and *trans*-products with a ratio of about 1:2. Alternatively, the Wittig-Horner reaction gave a satisfactory result, unexpectedly accompanied by the hydrolysis of methyl ester due to the strong basicity of *t*-BuOK. In addition, intermediate **10** was obtained conveniently through a routine method using NBS as the brominating agent and AIBN as the radical initiator. Since deprotection of the methyl of **12** in BBr_3_/DCM led to a mass of by-products, the reaction was carried out successfully in a mixture of AlCl_3_ and Et_3_N. Before the condensation of phenolic hydroxyl and the chlorinated intermediates **15c**~**30c**, the carboxyl group on the phenyl A ring of **13** was selectively protected by MeI. Slight methylation of the phenolic hydroxyl group occured in the same time.

Compounds **15c**~**30c** were synthesized from the corresponding methyl carboxylates **15a**~**30a** via reduction by LiAlH_4_ and chlorination with SOCl_2_ in DCM. Intermediates from the above two steps were used directly without any purification. Compounds **15a**, **16a** and **17a** were prepared according to the reported methods [23,24,25]. By reference to Kamal’s report, compounds **18a**~**30a** were synthesized from the corresponding substituted acetophenones [26].

### 2.2. In Vitro Biological Evaluation

All of the title compounds were assayed on the enzyme PTP1B in vitro. In compounds **15**~**18**, the phenyl position on the isoxazole ring caused huge differences in the inhibition against PTP1B. As shown in Table 1, both compounds **15** and **17**, characterized by a 3-phenyl-4-isoxazole moiety, exhibit moderate activities against the enzyme. Meanwhile, the 5-phenyl-4-isoxazole compound **16** (IC_50_ = 16.2 ± 4.52 μM) also has similar activity, while another 5-phenyl compound, **18**, exhibits a much higher activity (IC_50_ = 6.33 ± 1.02 μM) due to the different position of the isoxazole ring linked to the phenyl B ring. This result inspired us to further investigate the substituents on phenyl C and their effects on the activities against PTP1B. On the other hand, as expected, methylation of the carboxyl of **18** led to an obvious decrease on PTP1B inhibition.

Derivatives of **18** and their activities against PTP1B and TCPTP are listed in Table 2. For substituents at the 2-position of phenyl C, the electron-withdrawing chlorine atom (compound **19**) was beneficial to the activity when compared to an electron-donating methoxy (compound **20**). 3-subtituents also affect the activities remarkably. Compounds containing 3-Cl (**21**) and 3-Br (**23**) exhibit obvious inhibition to PTP1B, whereas a 3-F leads to a dramatic decrease in activity. NO_2_ (**25**, IC_50_ = 2.05 ± 0.54 μM) seems to be the optimal 3-substituent among the synthesized compounds, giving an activity 3-fold higher than compound that of **18**. In addition, when the 3-position is occupied by a methyl group, the resulting compound **24** still shows good inhibitory effect against the enzyme (IC_50_ = 3.68 ± 1.12 μM).

The derivatives present good tolerance of 4-substituents on the phenyl C ring, since compounds **26** (4-Me), **27** (4-OMe) and **28** (4-F) have similar activities against PTP1B. Amazingly, the di-substituted compounds, either dichloro (**29**) or difluoro (**30**) exhibited the highest inhibitory activities against the enzyme among all the title compounds. Most of all, compound **29** has an activity (IC_50_ = 0.91 ± 0.33 μM) about 14-fold higher than the lead compound LCA, which prompts us to further study on its structure modification and biological activity. However, as shown in Table 2, it is unfortunate that most of the compounds also exhibit some activities against TCPTP, except for compounds **20**, **22** and **23**. The selectivities against PTP1B and TCPTP vary a lot, from 1.4 to 22.9. Among the five compounds most potent against PTP1B (**19**, **25**, **27**, **29** and **30**), compound **19** has the best selectivity (TCPTP/PTP1B = 20.7) while compound **29** has about a 4-fold selectivity over TCPTP. These two compounds are promising for further study as anti-diabetes drugs.

### 2.3. Enzyme Kinetic Study

**Figure 3:**
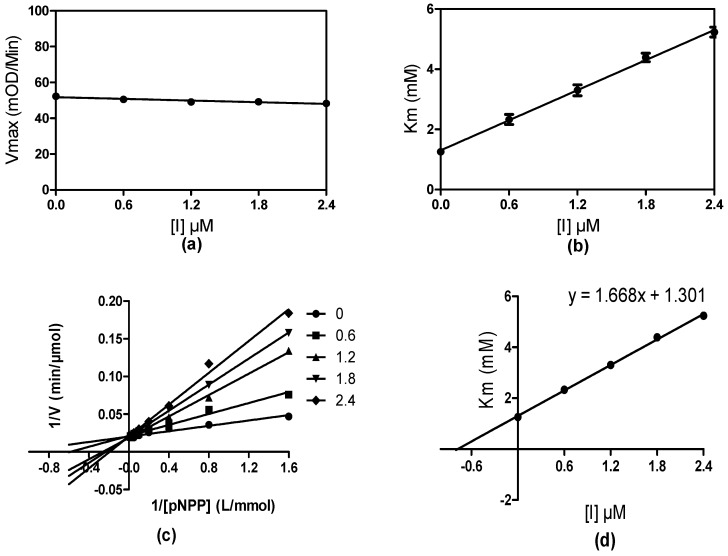
Characterization of **29** to PTP1B. (**a**,**b**) At various fixed concentrations of **29** the initial velocity was determined with various concentrations of pNPP; (**c**) Typical competitive inhibition of **29** shown by Lineweaver–Burk plot; (**d**) Linear replot of *K_m_* versus the inhibitor (**29**) concentration [I].

In order to determine the inhibition mode of the title compounds, the most active compound **29** was selected for a PTP1B enzyme kinetic study using the Michaelis-Menten equation. As shown in Figure 3, the *V*_max_ value remained constant while the *K*_m_ value increased with the mounting compound concentration, indicating that **29** was a competitive PTP1B inhibitor, which was further confirmed by a Lineweaver–Burk plot (Figure 3c). The calculated inhibitory constant *K*_i_ of compound **29** was 0.78 μM.

## 3. Experimental Section

### 3.1. Chemsitry

#### 3.1.1. General Information

All reagents were chemically pure and solvents were dried according to standard methods. The ^1^H-NMR and ^13^C-NMR spectra were obtained on an AV400 spectrometer (400 MHz, ^1^H; 100 MHz, ^13^C, Bruker, Billerica, MA, USA) in CDCl_3_ with tetramethylsilane as the internal standard. The melting points were determined on an X-4 binocular microscope melting point apparatus (Beijing Tech Instrument Co., Beijing, China) and are uncorrected. High-resolution mass data were obtained on a Micromass TOF II spectrometer (Micromass, Cary, NC, USA). The reactions were monitored by analytical thin-layer chromatography (TLC) carried out on silica gel GF_254_ plates with ultraviolet (UV) light detection.

#### 3.1.2. Synthesis of Methyl 3-((Diethoxyphosphoryl)methyl)benzoate (**11**)

A solution of methyl 3-methylbenzoate (47 g, 0.31 mol) in CCl_4_ (200 mL) was heated to reflux followed by addition of AIBN (2.6 g) in one portion. After that, NBS (67 g, 0.38 mol) was added carefully to the mixture during 2 h, and then the reaction was refluxed for an additional 5 h. After cooling to r.t. the mixture was filtered and evaporated under vacuum to give methyl 3-(bromomethyl)benzoate (**10**, 67 g, Yield: 94%) as a yellowish oil. The obtained oil was then dissolved in triethyl phosphate (130 g, 0.71 mol), heated to 160 °C and kept at this temperature for an additional 10 h. The mixture was concentrated on a rotary evaporator and then purified by flash chromatography (PE:EA = 5:1~PE:EA = 1:3) to give **11** as a yellow oil, which was used without any further purification.

#### 3.1.3. Synthesis of (*E*)-3-(4-Methoxystyryl)benzoic Acid (**12**)

To an ice-cooled solution of compound **11** (5 g, about 17.5 mmol) in THF (30 mL) was added a mixture of *t*-BuOK (3.9 g, 34.8 mmol) and THF (40 mL) during 30 min. After stirring for additional 30 min, a solution of 4-methoxybenzaldehyde (17.5 mmol) in THF (20 mL) was added and then the reaction mixture was warmed to room temperature, and stirred for another 8 h before quenching with 2 N HCl. The mixture was then evaporated to remove the THF and precipitation occurred meanwhile. The precipitate was then filtered and dried to give **12** as a yellow solid without any additional purification. Yield: 4.8 g (46%); ^1^H-NMR (DMSO-*d*_6_) δ: 12.93 (brs, 1H), 8.12 (s, 1H), 7.81 (d, *J* = 7.6 Hz, 2H), 7.58 (d, *J* = 8.6 Hz, 2H), 7.49 (t, *J* = 7.8 Hz, 1H), 7.28 (d, *J* = 16.5 Hz, 1H), 7.18 (d, *J* = 16.5 Hz, 1H), 6.96 (d, *J* = 8.6 Hz, 2H), 3.78 (s, 3H).

#### 3.1.4. Synthesis of (*E*)-3-(4-Hydroxystyryl)benzoic Acid (**13**)

Anhydrous aluminium trichloride (30 g) was added to triethylamine (200 mL) in portions during 30 min and then heated to 60 °C for 3 h before adding compound **12** (16.5 g, 65.0 mmol) in one portion. The resultant mixture was then heated to 80 °C and kept for another 16 h. After evaporating most of the triethylamine, the reaction was quenched by addition of iced hydrochloric acid. The water phase was extracted by ethyl acetate (80 mL × 3), dried over anhydrous Na_2_SO_4_, filtered and then evaporated to give crude **13**, which was then recrystallized from ethyl acetate to give **13** as a yellowish solid. Yield: 7.8 g (48%); ^1^H-NMR (DMSO-*d*_6_) δ: 12.98 (brs, 1H), 9.63 (s, 1H), 8.09 (s, 1H), 7.78~7.81 (m, 2H), 7.48~7.49 (m, 3H), 7.13~7.15 (m, 2H), 6.78 (d, *J* = 8.7 Hz, 2H).

#### 3.1.5. Synthesis of Methyl (*E*)-3-(4-Hydroxystyryl)benzoate (**14**)

Anhydrous K_2_CO_3_ (0.58 g, 4.2 mmol) was added to a solution of **13** (1.68 g, 7 mmol) in acetone (15 mL), and the mixture was then stirred for 2 h at room temperature before adding MeI (1.07 g, 4.2 mmol) in one portion. The reaction was then stirred for an additional 16 h. After that, the mixture was filtered, evaporated and purified by column chromatography (PE:EA = 3:1) to give **14** as a yellowish solid. Yield: 0.29 g (51%); ^1^H-NMR (DMSO-*d*_6_) δ: 9.64 (s, 1H), 8.10 (s, 1H), 7.79~7.85 (m, 2H), 7.46~7.50 (m, 3H), 7.25 (d, *J* = 16.5 Hz, 1H), 7.12 (d, *J* = 16.5 Hz, 1H), 6.78 (d, *J* = 8.5 Hz, 2H), 3.88 (s, 3H); ^13^C-NMR (DMSO-*d*_6_) δ: 166.2, 157.6, 138.2, 130.3, 130.1, 129.7, 129.1, 128.1 (2C), 127.7, 127.4, 126.6, 123.9, 115.5 (2C), 52.1.

#### 3.1.6. Synthesis of (3-Phenylisoxazol-4-yl)methanol (**15b**)

On an ice bath, to a solution of **15a** (5 mmol) in anhydrous THF (20 mL) was added LiAlH_4_ (5 mmol) in portions during 30 min. After that, the mixture was stirred for 2 h and quenched by 1 mL H_2_O added dropwise. After adding anhydrous Na_2_SO_4_ (5 g), the reaction was filtered and evaporated to give **15b**, which was used in the next step without any purification. Intermediates **16b**~**30b** were synthesized according to methods described above for the synthesis of **15b**.

#### 3.1.7. Synthesis of 4-(Chloromethyl)-3-phenylisoxazole (**15c**)

To a solution of **15b** (3 mmol), DCM (20 mL) and DMF (1~2 drops), SOCl_2_ (10 mmol) was added and heated to 45 °C. After keeping at this temperature for 8 h, the solution was evaporated to give **15c** as yellow oil, which was used in the next step without any purification. Intermediates **16c**~**30c** were synthesized according to the above method for the synthesis of **15c** and purified by column chromatography (PE:EA = 5:1~10:1) if necessary.

#### 3.1.8. Synthesis of (*E*)-3-(4-((3-Phenylisoxazol-4-yl)methoxy)styryl)benzoic Acid (**15**)

A mixture of **14** (1 mmol), **15c** (1 mmol) and Cs_2_CO_3_ (2 mmol) in DMSO (5 mL) was heated to 85 °C and kept at this temperature for 8 h, then the reaction mixture was cooled to 60 °C followed by the addition of 20% KOH solution (2 mL). After stirring at 60 °C for 4 h, the reaction mixture was cooled, acidified by 1 N hydrochloric acid diluted with water to give a mass of precipitate of the crude product, which was then recrystallized from methanol to give the purified **15**. Yellow solid; Yield: 63%; ^1^H-NMR (DMSO-*d*_6_) δ: 13.12 (brs, 1H), 9.22 (s, 1H), 8.07 (s, 1H), 7.77~7.79 (m, 2H), 7.48~7.59 (m, 7H), 7.27 (t, *J* = 7.6 Hz, 1H), 7.18 (d, *J* = 16.5 Hz, 1H), 7.13 (d, *J* = 16.5 Hz, 1H), 7.04 (d, *J* = 8.8 Hz, 2H), 5.13 (s, 2H); ^13^C-NMR (DMSO-*d*_6_) δ: 169.2, 161.3, 159.2, 157.7, 136.7, 131.0, 130.6, 129.6 (2C), 129.4, 128.7, 128.6, 128.4 (2C), 128.3 (2C), 128.0, 127.8, 127.4, 127.3, 115.6 (2C), 114.6, 59.1; HRMS (ESI) calcd for C_25_H_18_NO_4_ [M − H]^−^ 396.1236, found 396.1265 (see Supplementary Materials). Compounds **16**~**30** were synthesized according to the above method for the synthesis of **15**.

*(E)-3-(4-((5-Phenylisoxazol-4-yl)methoxy)styryl)benzoic acid* (**16**): Yellow solid; Yield: 47%; ^1^H-NMR (DMSO-*d*_6_) δ: 13.15 (brs, 1H), 9.22 (s, 1H), 8.13 (s, 1H), 7.77~7.80 (m, 4H), 7.60 (d, *J* = 8.7 Hz, 2H), 7.52~7.54 (m, 4H), 7.31 (d, *J* = 16.5 Hz, 1H), 7.22 (d, *J* = 16.5 Hz, 1H), 7.07 (d, *J* = 8.7 Hz, 2H), 5.14 (s, 2H); ^13^C-NMR (DMSO-*d*_6_) δ: 169.5, 161.3, 159.1, 158.1, 138.4, 131.0, 130.6, 130.5, 129.7, 129.6, 129.5 (2C), 129.4, 128.6 (2C), 128.4 (2C), 128.2, 127.8, 127.3, 125.9, 115.6 (2C), 114.6, 59.1; HRMS (ESI) calcd for C_25_H_18_NO_4_ [M − H]^−^ 396.1236, found 396.1212.

*(E)-3-(4-((5-Methyl-3-phenylisoxazol-4-yl)methoxy)styryl)benzoic acid* (**17**): Yellow solid; Yield: 42%; ^1^H-NMR (DMSO-*d*_6_) δ: 13.04 (brs, 1H), 8.13 (s, 1H), 7.77 (d, *J* = 7.2 Hz, 1H), 7.69~7.72 (m, 2H), 7.62 (d, *J* = 7.8 Hz, 1H), 7.58 (d, *J* = 8.6 Hz, 1H), 7.49~7.52 (m, 3H), 7.36 (t, *J* = 7.7 Hz, 1H), 7.23 (d, *J* = 16.5 Hz, 1H), 7.12 (d, *J* = 16.5 Hz, 1H), 7.02 (s, 1H), 7.10 (d, *J* = 8.6 Hz, 2H), 5.00 (s, 2H), 2.53 (s, 3H); ^13^C-NMR (DMSO-*d*_6_) δ: 170.4, 168.3, 162.6, 158.1, 137.3, 130.9, 130.4, 129.5 (2C), 129.1, 129.0, 128.7, 128.6, 128.4, 128.3 (2C), 128.2 (2C), 127.4, 126.9, 115.7 (2C), 109.9, 59.5, 11.3; HRMS (ESI) calcd for C_26_H_20_NO_4_ [M − H]^−^ 410.1392, found 410.1388.

*(E)-3-(4-((5-Phenylisoxazol-3-yl)methoxy)styryl)benzoic acid* (**18**): Yellow solid; Yield: 42%; ^1^H-NMR (DMSO-*d*_6_) δ: 13.15 (brs, 1H), 8.12 (s, 1H), 7.90 (dd, *J*_1_ = 8.0 Hz, *J*_2_ = 1.8 Hz, 2H), 7.80 (t, *J* = 7.5 Hz, 2H), 7.62 (d, *J* = 8.8 Hz, 2H), 7.52~7.58 (m, 3H), 7.47 (t, *J* = 7.7 Hz, 1H), 7.29 (d, *J* = 16.4 Hz, 1H), 7.22 (d, *J* = 16.4 Hz, 1H), 7.20 (s, 1H), 7.10 (d, *J* = 8.8 Hz, 2H), 5.29 (s, 2H); ^13^C-NMR (DMSO-*d*_6_) δ: 170.0, 168.2, 161.8, 158.1, 138.0, 131.1, 130.8, 130.3, 129.8 (2C), 129.3, 129.2, 128.5 (2C), 128.4, 127.4, 127.1, 126.3, 126.1 (2C), 115.6 (2C), 100.5, 61.8; HRMS (ESI) calcd for C_25_H_18_NO_4_ [M − H]^−^ 396.1236, found 396.1219.

*(E)-3-(4-((5-(2-Chlorophenyl)isoxazol-3-yl)methoxy)styryl)benzoic acid* (**19**): Yellow solid; Yield: 51%; ^1^H-NMR (DMSO-*d*_6_) δ: 13.02 (s, 1H), 8.12 (s, 1H), 7.99 (d, *J* = 8.4 Hz, 1H), 7.97 (d, *J* = 5.5 Hz, 1H), 7.78~7.85 (m, 3H), 7.62 (d, *J* = 8.4 Hz, 2H), 7.46~7.51 (m, 2H), 7.40 (t, *J* = 8.7 Hz, 2H), 7.30 (d, *J* = 16.4 Hz, 1H), 7.22 (d, *J* = 16.4 Hz, 1H), 7.18 (s, 1H), 7.09 (d, *J* = 8.4 Hz, 2H), 5.28 (s, 2H); ^13^C-NMR (DMSO-*d*_6_) δ: 168.6, 167.3, 161.3, 157.6, 137.7, 131.2, 130.2, 128.9, 128.2, 128.1, 128.1, 128.0 (2C), 127.9, 126.9, 124.1, 125.6, 116.5, 116.3, 115.5, 115.1 (2C), 99.9, 61.3; HRMS (ESI) calcd for C_25_H_17_ClNO_4_ [M − H]^−^ 430.0846, found 430.0831.

*(E)-3-(4-((5-(2-Methoxyphenyl)isoxazol-3-yl)methoxy)styryl)benzoic acid* (**20**): Yellow solid; Yield: 47%; ^1^H-NMR (DMSO-*d*_6_) δ: 13.05 (brs, 1H), 8.13 (s, 1H), 7.79~7.83 (m, 2H), 7.62 (d, *J* = 8.8 Hz, 2H), 7.46~7.52 (m, 3H), 7.26 (d, *J* = 16.4 Hz, 1H), 7.20 (d, *J* = 16.4 Hz, 1H), 7.10~7.14 (m, 2H), 6.99 (s, 1H), 6.79 (d, *J* = 8.8 Hz, 2H), 5.29 (s, 2H), 3.96 (s, 3H); ^13^C-NMR (DMSO-*d*_6_) δ: 167.8, 166.2, 161.5, 158.2, 138.4, 138.2, 131.7, 130.7, 130.5, 130.0, 129.4, 129.4, 128.6 (2C), 128.2, 127.4, 127.2, 126.1, 124.6, 121.3, 115.6 (2C), 103.6, 61.8, 56.3; HRMS (ESI) calcd for C_26_H_20_NO_5_ [M − H]^−^ 426.1341, found 426.1376.

*(E)-3-(4-((5-(3-Chlorophenyl)isoxazol-3-yl)methoxy)styryl)benzoic acid* (**21**): Yellow solid; Yield: 40%; ^1^H-NMR (DMSO-*d*_6_) δ: 13.12 (brs, 1H), 8.13 (s, 1H), 8.01 (s, 1H), 7.78~7.82 (m, 2H), 7.62 (d, *J* = 8.6 Hz, 2H), 7.58~7.59 (m, 2H), 7.46~7.50 (m, 2H), 7.33 (s, 1H), 7.30 (d, *J* = 16.4 Hz, 1H), 7.22 (d, *J* = 16.4 Hz, 1H), 7.10 (d, *J* = 8.6 Hz, 2H), 5.30 (s, 2H); ^13^C-NMR (DMSO-*d*_6_) δ: 168.5, 167.9, 161.9, 158.1, 138.2, 134.5, 131.7, 130.8, 130.6, 130.0, 129.4, 129.0, 128.5, 127.4, 126.2 (2C), 125.8, 124.7, 124.6, 115.6 (2C), 101.7; HRMS (ESI) calcd for C_25_H_17_ClNO_4_ [M − H]^−^ 430.0846, found 430.0857.

*(E)-3-(4-((5-(3-Fluorophenyl)isoxazol-3-yl)methoxy)styryl)benzoic acid* (**22**): Yellow solid; Yield: 48%; ^1^H-NMR (DMSO-*d*_6_) δ: 13.05 (brs, 1H), 8.13 (s, 1H), 7.76~7.84 (m, 4H), 7.58~7.64 (m, 3H), 7.49 (t, *J* = 7.7 Hz, 1H), 7.29~7.40 (m, 3H), 7.22 (d, *J* = 16.4 Hz, 1H), 7.10 (d, *J* = 8.7 Hz, 2H), 5.30 (s, 2H); ^13^C-NMR (DMSO-*d*_6_) δ: 168.7, 167.7, 162.9 (d, *J* = 145.6 Hz), 161.9, 158.1, 138.2, 132.1, 132.0, 131.7, 130.7, 129.4, 129.1, 129.0, 128.5 (2C), 128.4, 127.4, 126.2, 122.3, 118.2 (d, *J* = 21.2 Hz), 115.6 (2C), 113.0 (d, *J* = 23.7 Hz), 101.6, 61.7; HRMS (ESI) calcd for C_25_H_17_FNO_4_ [M − H]^−^ 414.1142, found 414.1133.

*(E)-3-(4-((5-(3-Bromophenyl)isoxazol-3-yl)methoxy)styryl)benzoic acid* (**23**): Yellow solid; Yield: 51%; ^1^H-NMR (DMSO-*d*_6_) δ: 13.13 (brs, 1H), 8.14 (s, 1H), 8.12 (s, 1H), 7.89~7.93 (m, 1H), 7.79 (d, *J* = 7.6 Hz, 1H), 7.71~7.73 (m, 1H), 7.61 (d, *J* = 8.7 Hz, 2H), 7.49~7.58 (m, 2H), 7.43 (t, *J* = 7.6 Hz, 1H), 7.27 (d, *J* = 16.5 Hz, 1H), 7.20 (d, *J* = 16.5 Hz, 1H), 7.19 (s, 1H), 7.09 (d, *J* = 8.7 Hz, 2H), 5.29 (s, 2H); ^13^C-NMR (DMSO-*d*_6_) δ:170.0, 168.4, 161.8, 158.0, 137.8, 133.7, 131.9, 131.0, 130.9, 129.8, 129.1, 128.9, 128.7, 128.5 (2C), 127.4, 126.6, 126.1, 125.0, 123.0, 115.6 (2C), 100.5, 61.7; HRMS (ESI) calcd for C_25_H_17_BrNO_4_ [M − H]^−^ 474.0341, found 474.0371.

*(E)-3-(4-((5-(m-Tolyl)isoxazol-3-yl)methoxy)styryl)benzoic acid* (**24**): Yellow solid; Yield: 44%; ^1^H-NMR (DMSO-*d*_6_) δ: 13.05 (brs, 1H), 8.12 (s, 1H), 7.96 (s, 1H), 7.80 (t, *J* = 7.0 Hz, 2H), 7.61~7.73 (m, 4H), 7.41~7.49 (m, 2H), 7.33 (d, *J* = 16.5 Hz, 1H), 7.25 (d, *J* = 16.5 Hz, 1H), 7.41 (s, 1H), 7.10 (d, *J* = 8.8 Hz, 2H), 5.28 (s, 2H), 2.39 (s, 3H); ^13^C-NMR (DMSO-*d*_6_) δ: 170.2, 168.1, 161.7, 158.1, 139.2, 138.0, 131.7, 130.7, 130.3, 129.7, 129.3, 129.2, 128.5 (2C), 128.4, 127.4, 127.0, 126.5, 126.3, 123.3, 115.6 (2C), 100.4, 61.8, 21.3; HRMS (ESI) calcd for C_26_H_20_NO_4_ [M − H]^−^ 410.1392, found 410.1388.

*(E)-3-(4-((5-(3-Nitrophenyl)isoxazol-3-yl)methoxy)styryl)benzoic acid* (**25**): Yellow solid; Yield: 38%; ^1^H-NMR (DMSO-*d*_6_) δ: 13.17 (brs, 1H), 8.35 (s, 1H), 8.13 (s, 1H), 7.78~7.83 (m, 3H), 7.63 (d, *J* = 8.8 Hz, 2H), 7.46~7.49 (m, 4H), 7.28 (d, *J* = 16.5 Hz, 1H), 7.22 (d, *J* = 16.5 Hz, 1H), 7.11 (d, *J* = 8.8 Hz, 2H), 5.33 (s, 2H); ^13^C-NMR (DMSO-*d*_6_) δ: 167.7, 162.2, 158.0, 148.9, 138.5, 138.2, 131.7, 130.7, 130.5, 130.0, 129.4, 128.6 (2C), 128.4, 127.4, 127.2, 126.2, 125.4, 124.6, 120.7, 115.6 (2C), 102.5, 61.7; HRMS (ESI) calcd for C_25_H_17_N_2_O_6_ [M − H]^−^ 441.1087, found 441.1072.

*(E)-3-(4-((5-(p-Tolyl)isoxazol-3-yl)methoxy)styryl)benzoic acid* (**26**): Yellow solid; Yield: 55%; ^1^H-NMR (DMSO-*d*_6_) δ: 12.98 (brs, 1H), 8.13 (s, 1H), 7.82~7.88 (m, 2H), 7.79 (d, *J* = 8.1 Hz, 1H), 7.62 (d, *J* = 8.6 Hz, 2H), 7.49~7.54 (m, 1H), 7.35 (d, *J* = 8.1 Hz, 2H), 7.47 (t, *J* = 7.7 Hz, 1H), 7.28 (d, *J* = 16.5 Hz, 1H), 7.23 (d, *J* = 16.5 Hz, 1H), 7.10 (d, *J* = 8.6 Hz, 2H), 7.09 (s, 1H), 5.27 (s, 2H), 2.37 (s, 3H); 170.2, 166.7, 161.7, 158.2, 141.0, 138.4, 131.0, 130.6, 130.3 (2C), 129.7, 129.6, 128.6 (2C), 127.3, 126.1 (2C), 125.9, 124.4, 115.6 (2C), 99.9, 61.8, 21.5; HRMS (ESI) calcd for C_26_H_20_NO_4_ [M − H]^−^ 410.1392, found 410.1373.

*(E)-3-(4-((5-(4-Methoxyphenyl)isoxazol-3-yl)methoxy)styryl)benzoic acid* (**27**): Yellow solid; Yield: 52%; ^1^H-NMR (DMSO-*d*_6_) δ: 13.04 (brs, 1H), 8.12 (s, 1H), 7.77~7.86 (m, 5H), 7.62 (d, *J* = 8.4 Hz, 2H), 7.46~7.54 (m, 2H), 7.30 (d, *J* = 16.5 Hz, 1H), 7.22 (d, *J* = 16.5 Hz, 1H), 7.09 (d, *J* = 8.4 Hz, 2H), 7.04 (s, 1H), 5.26 (s, 2H), 3.83 (s, 3H); ^13^C-NMR (DMSO-*d*_6_) δ: 167.7, 166.1, 161.5, 158.2, 138.4, 138.2, 131.7, 130.7, 130.7, 130.0, 129.4, 128.6 (2C), 127.7, 127.5, 127.4, 127.2, 126.1, 124.6, 120.0, 115.2 (2C), 100.0, 61.8, 55.7; HRMS (ESI) calcd for C_26_H_20_NO_5_ [M − H]^−^ 426.1341, found 426.1365.

*(E)-3-(4-((5-(4-Fluorophenyl)isoxazol-3-yl)methoxy)styryl)benzoic acid* (**28**): Yellow solid; Yield: 37%; ^1^H-NMR (DMSO-*d*_6_) δ: 13.10 (brs, 1H), 8.13 (s, 1H), 7.83~7.95 (m, 5H), 7.64 (d, *J* = 8.5 Hz, 2H), 7.48~7.55 (m, 2H), 7.31 (d, *J* = 16.5 Hz, 1H), 7.24 (d, *J* = 16.5 Hz, 1H), 7.21 (s, 1H), 7.11 (d, *J* = 8.4 Hz, 2H), 5.33 (s, 2H); ^13^C-NMR (DMSO-*d*_6_) δ: 168.1, 166.7, 161.5, 158.8 (d, *J* = 154.0 Hz, 2C), 158.1, 139.4, 132.5, 132.4 (2C), 130.9,129.9, 128.7 (2C), 128.5, 128.2, 127.9, 127.3, 126.2 (d, *J* = 26.5 Hz, 2C), 124.6, 115.5 (2C), 104.8, 61.8; HRMS (ESI) calcd for C_25_H_17_FNO_4_ [M − H]^−^ 414.1142, found 414.1159.

*(E)-3-(4-((5-(3,4-Dichlorophenyl)isoxazol-3-yl)methoxy)styryl)benzoic acid* (**29**): Yellow solid; Yield: 52%; ^1^H-NMR (DMSO-*d*_6_) δ: 13.03 (brs, 1H), 8.22 (s, 1H), 8.12 (s, 1H), 7.80~7.83 (m, 3H), 7.62 (d, *J* = 8.7 Hz, 2H), 7.45~7.52 (m, 2H), 7.37 (s, 1H), 7.30 (d, *J* = 16.4 Hz, 1H), 7.23 (d, *J* = 16.4 Hz, 1H), 7.09 (d, *J* = 8.7 Hz, 2H), 5.30 (s, 2H); ^13^C-NMR (DMSO-*d*_6_) δ: 167.3, 167.1, 161.5, 157.5, 137.7, 133.1, 132.2, 131.6, 131.3, 130.5, 130.3, 130.2, 130.1, 129.1, 128.9, 128.1, 128.0 (2C), 127.5, 127.0, 126.9, 125.7, 115.5, 115.1 (2C), 101.6, 61.2; HRMS (ESI) calcd for C_25_H_16_Cl_2_NO_4_ [M − H]^−^ 464.0456, found 464.0438.

*(E)-3-(4-((5-(3,4-Difluorophenyl)isoxazol-3-yl)methoxy)styryl)benzoic acid* (**30**): Yellow solid; Yield: 45%; ^1^H-NMR (DMSO-*d*_6_) δ: 13.09 (brs, 1H), 8.10 (s,1H), 8.03~8.08 (m, 1H), 7.72~7.80 (m, 3H), 7.57~7.67 (m, 3H), 7.43 (t, *J* = 7.7 Hz, 1H), 7.17~7.29 (m, 3H), 7.08 (d, *J* = 8.7 Hz, 1H), 6.96 (d, *J* = 8.5 Hz, 1H), 5.29 (s, 2H); ^13^C-NMR (DMSO-*d*_6_) δ: 167.3, 167.2, 159.1, 157.5, 137.9, 137.7, 130.7 (d, *J* = 97.3 Hz), 130.6 (d, *J* = 97.0 Hz), 130.0, 129.5, 128.9, 128.1, 128.0, 127.7, 127.6, 126.9, 126.7, 125.6, 124.1, 115.5, 115.1, 100.9, 61.2; HRMS (ESI) calcd for C_25_H_16_F_2_NO_4_ [M − H]^−^ 432.1047, found 432.1083.

### 3.2. Biological Activity against PTP1B and TCPTP

A colorimetric assay to measure inhibition against PTP1B and TCPTP was performed in 96-well plates. The assay was conducted as described by Zhang et al. [27]. Briefly, the tested compounds were solubilized in DMSO and serially diluted into concentrations for the inhibitory test. The assays were carried out in a final volume of 100 mL containing 50 mmol/L MOPS, pH 6.5, 2 mmol/L pNPP, 30 nmol/L GST-PTP1B or GST-TCPTP, and 2% DMSO, and the catalysis of pNPP was continuously monitored on a SpectraMax 340 microplate reader (Molecular Devices, Sunnyvale, CA, USA) at 405 nm for 2 min at 30 °C. The IC_50_ value was calculated from the nonlinear curve fitting of the percent inhibition [inhibition (%)] vs. the inhibitor concentration [I] using the following equation:

Inhibition (%) = 100/[1 + (IC_50_/[I])·κ],

where κ is the Hill coefficient.

### 3.3. Characterization of Compound ***29***

To determine the mode of inhibition of compound **29**, an assay was carried out in a 100 μL assay mixture containing 50 mM MOPS at pH 6.5, 30 nM PTP1B, pNPP in 2-fold dilution up to 80 mM, and different concentrations of inhibitor as described by Zhang et al. [27]. In the presence of the competitive inhibitor, the Michaelis–Menten equation is described as 1/*ν* = (*K*_m_/[*V*_max_[S]])·(1 + [I]/*K*_i_) + 1/*V*_max_, where *ν* is the initial rate, *V*_max_ is the maximum rate, and [S] is the substrate concentration. *K*_i_ value was obtained by Lineweaver–Burk plot of apparent *K*_m_/*V*_max_ (slope) from primary reciprocal plot versus the inhibitor concentration [I] according to the equation *K*_m_/*V*_max_ = 1 + [I]/*K*_i_.

## 4. Conclusions

In this paper we report a series of stilbene derivatives containing phenyl-substituted isoxazoles as inhibitors against protein tyrosine phosphatase 1B (PTP1B). Most of these compounds showed moderate to good inhibitory activity against PTP1B. The most potent compound **29** (IC_50_ = 0.91 ± 0.33 μM) showed 14-fold improved PTP1B inhibitory activity compared to the lead compound LCA and about a 4-fold selectivity over TCPTP. Our SAR study clarified the effects of the substituents on the phenyl C ring on the corresponding activities. Compound **29** was identified as a competitive inhibitor of PTP1B with a *K*_i_ value of 0.78 μM in enzyme kinetic studies. These novel stilbene derivatives could be promising lead compounds for the development of a new class of PTP1B inhibitors. Further SAR research and evaluation of new derivatives are still in progress.

## Figures and Tables

**Table 1:**
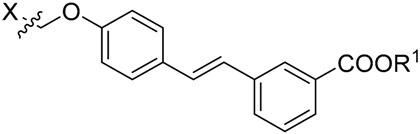
The structures of compounds **15**, **16**, **17**, **18**, **18d** and their IC_50_ values against PTP1B.

Compound	X	R^1^	IC_50_ (μM) ^a^
**15**	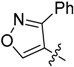	H	20.7 ± 3.83
**16**	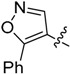	H	16.2 ± 4.52
**17**	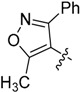	H	15.7 ± 2.15
**18**	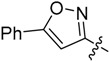	H	6.33 ± 1.02
**18d**	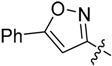	Me	23.7 ± 3.62
Oleanolic acid ^b^			2.96 ± 0.35

^a^ IC_50_ values were determined by regression analyses and expressed as means ± SD of three replications; ^b^ Positive control.

**Table 2:**
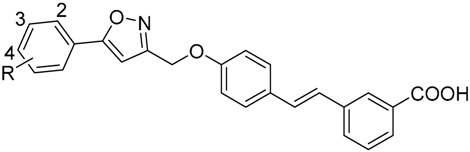
The structures of compounds **18**~**30** and their IC_50_ values against PTP1B and TCPTP.

Compound	R	IC_50_ (μM) ^a^		TCPTP/PTP1B ^b^
		PTP1B	TCPTP	
**18**	H	6.33 ± 1.33	9.58 ± 0.33	1.5
**19**	2-Cl	1.75 ± 0.82	36.15 ± 3.82	20.7
**20**	2-OMe	7.66 ± 1.05	>40	5.3
**21**	3-Cl	5.37 ± 1.53	7.58 ± 1.32	1.4
**22**	3-F	>20	>40	- ^c^
**23**	3-Br	9.85 ± 2.04	>40	4.1
**24**	3-Me	3.68 ± 1.12	18.91 ± 1.18	5.1
**25**	3-NO_2_	2.05 ± 0.54	13.25 ± 1.70	6.5
**26**	4-Me	3.56 ± 0.28	7.67 ± 0.35	2.2
**27**	4-OMe	2.23 ± 0.51	8.02 ± 0.99	3.6
**28**	4-F	3.34 ± 0.81	5.19 ± 0.31	1.6
**29**	3,4-Cl_2_	0.91 ± 0.33	3.78 ± 0.22	4.2
**30**	3,4-F_2_	1.22 ± 0.48	6.02 ± 1.13	4.9
Lithocholic acid		12.54 ± 2.51	20.95 ± 3.66	1.7
Oleanolic acid ^d^		2.71 ± 0.19	6.12 ± 0.15	2.3

^a^ IC_50_ values were determined by regression analyses and expressed as means ± SD of three replications; ^b^ TCPTP/PTP1B, the ratio of IC_50_ of TCPTP and PTP1B; ^c^ “-”, none accurate value was calculated; ^d^ Positive control.

## Supplementary Materials

Supplementary materials can be accessed at: http://www.mdpi.com/1420-3049/21/12/1722/s1.
