# Supplementary material for: Design, Synthesis and Biological Evaluation of Stilbene Derivatives as Novel Inhibitors of Protein Tyrosine Phosphatase 1B
Source: Molecules. 2016 Dec 16;21(12):1722. doi: 10.3390/molecules21121722 (PMC6274251; doi:10.3390/molecules21121722)
Supplement: Supplementary file 1 [file molecules-21-01722-s001.pdf]

Supplementary Materials: Design, Synthesis and Biological Evaluation of Stilbene Derivatives as Novel Inhibitors of Protein Tyrosine Phosphatase 1B

Haibing He, Yinghua Ge, Hong Dai, Song Cui, Fei Ye, Jia Jin and Yujun Shi

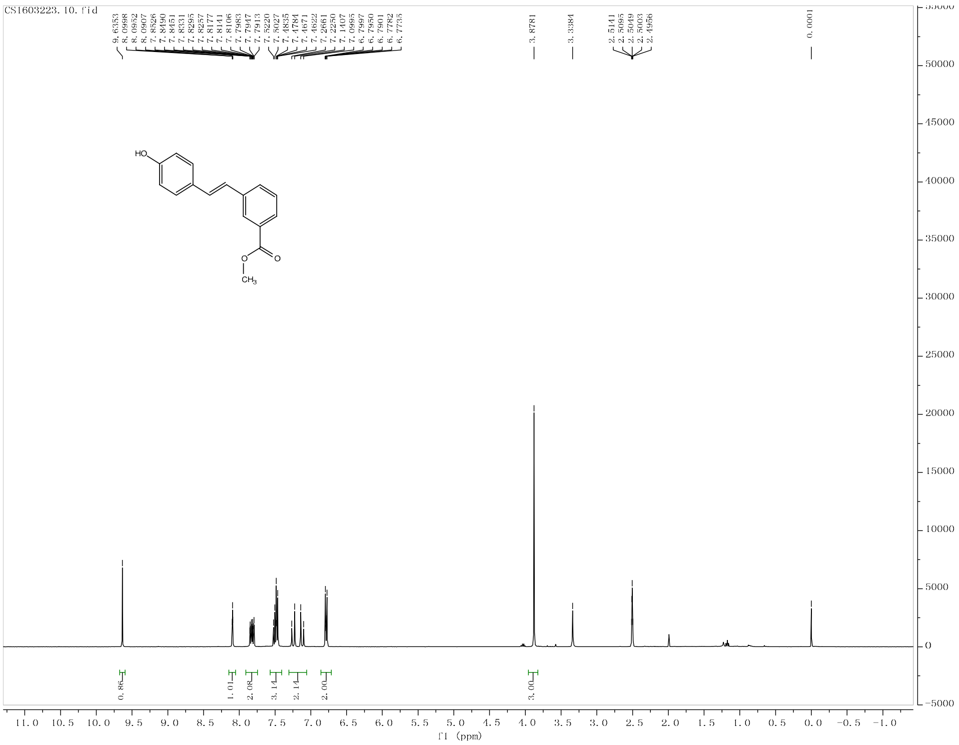

Figure S1. ^{1}H-NMR spectrum of compound **14**.

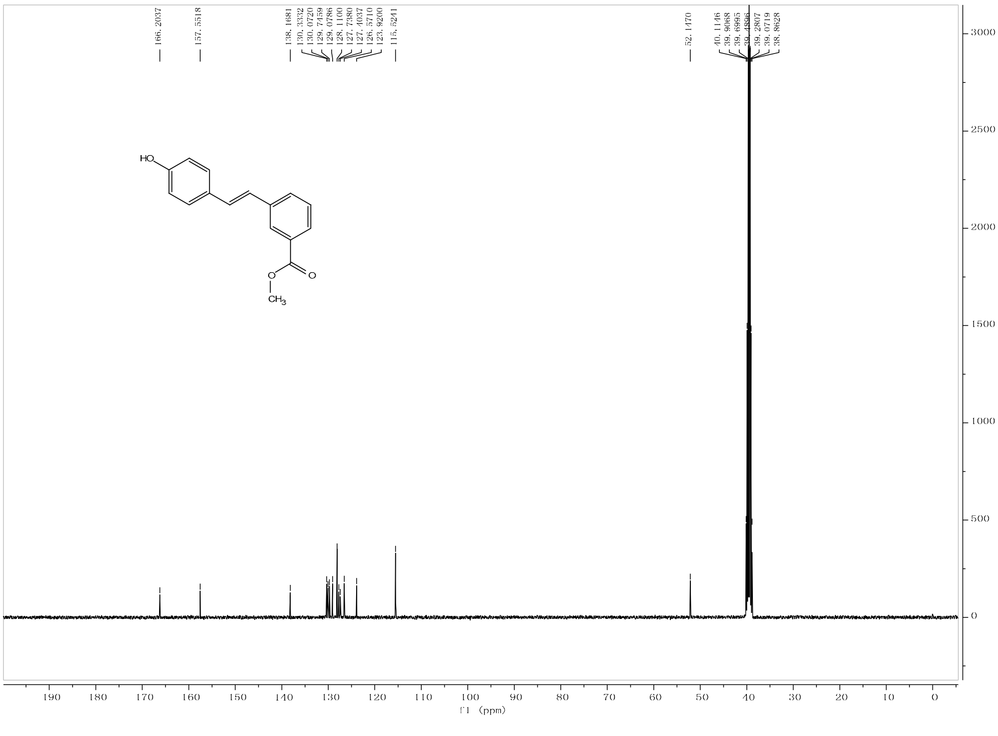

Figure S2. ^{13}C-NMR spectrum of compound **14**.

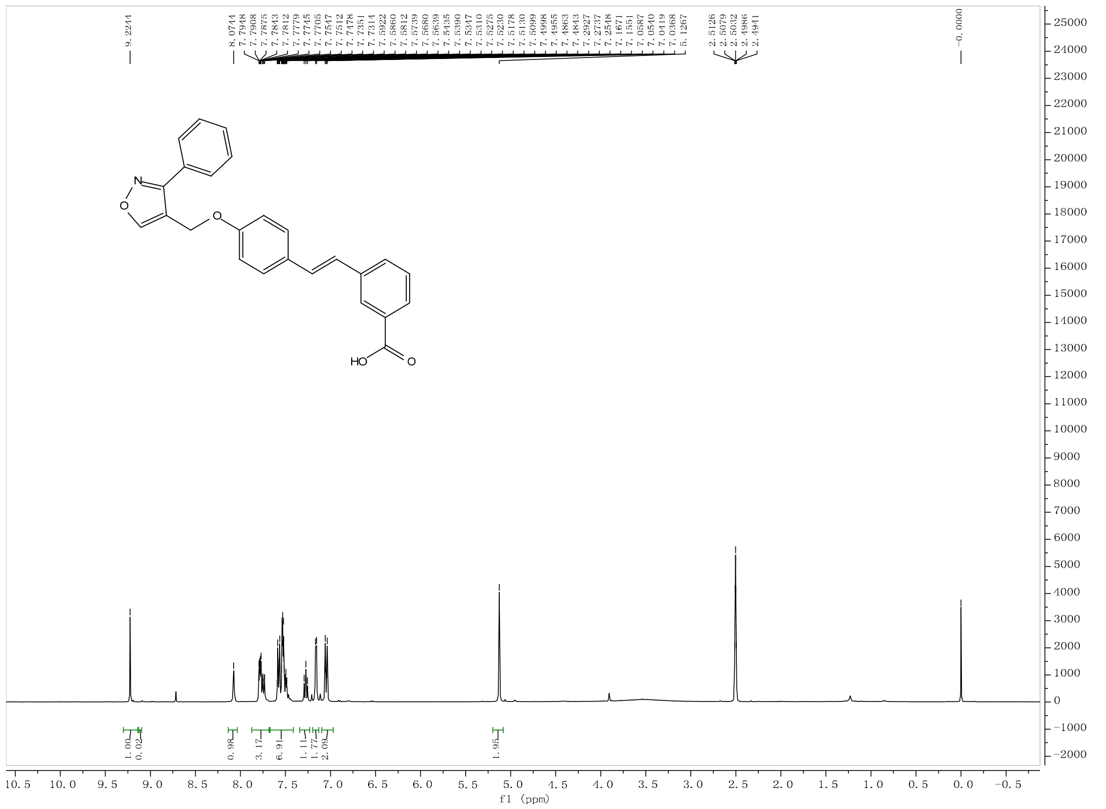

Figure S3. ^{1}H-NMR spectrum of compound **15**.

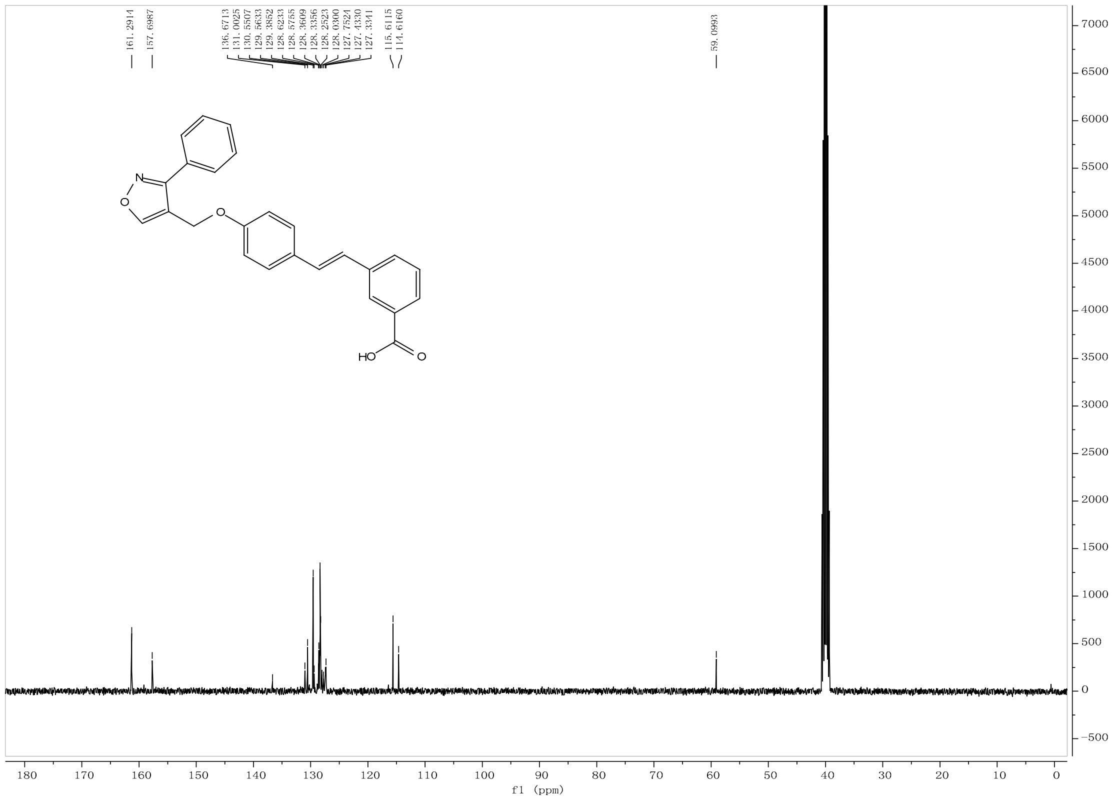

Figure S4. ^{13}C-NMR spectrum of compound **15**.

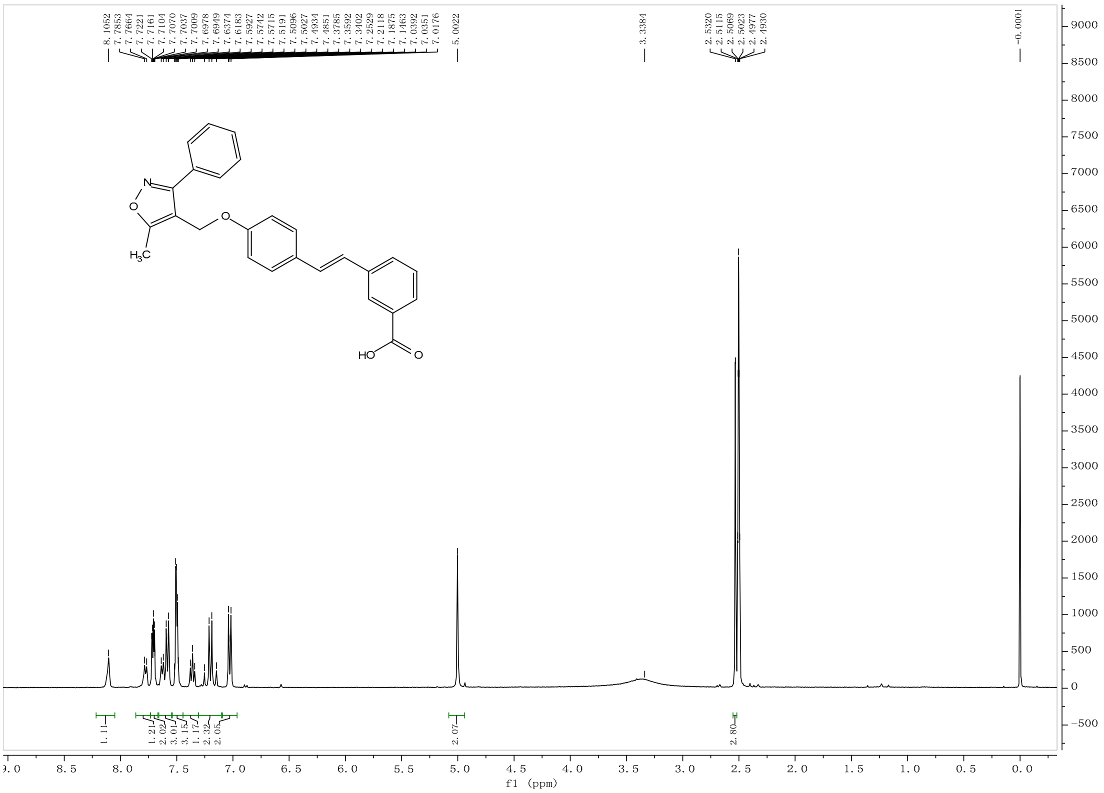

Figure S5. ^{1}H-NMR spectrum of compound **17**.

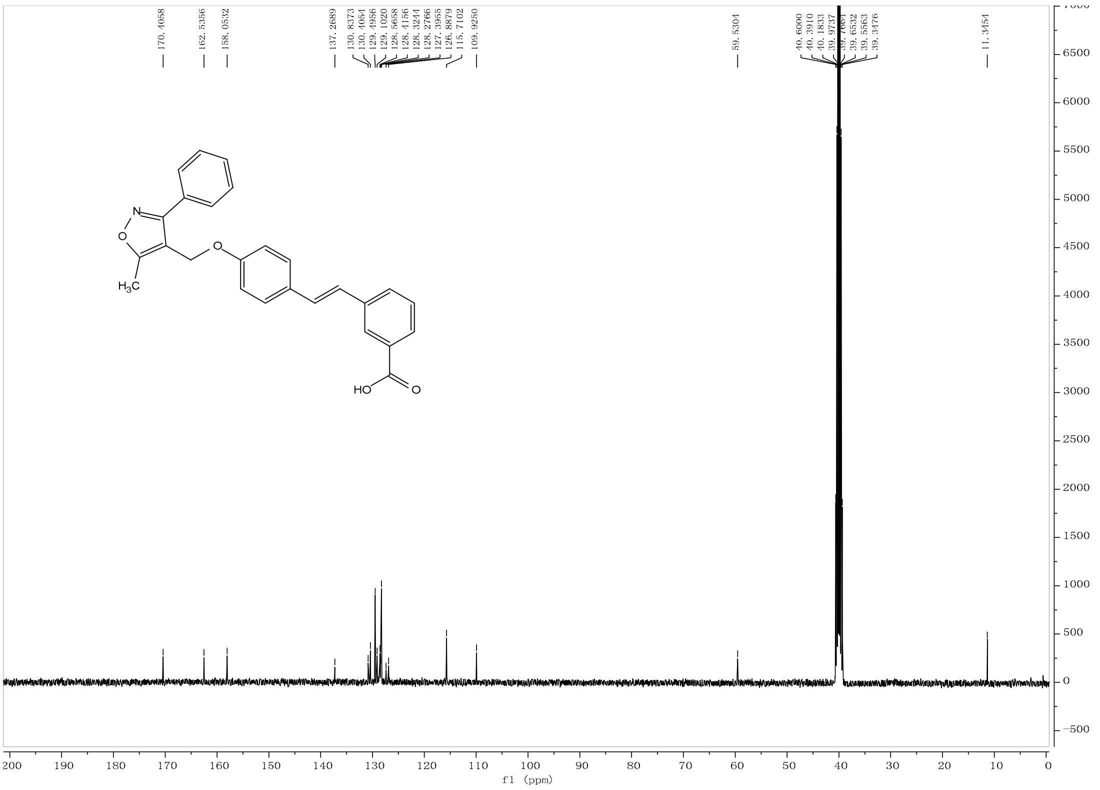

Figure S6. ^{13}C-NMR spectrum of compound **17**.

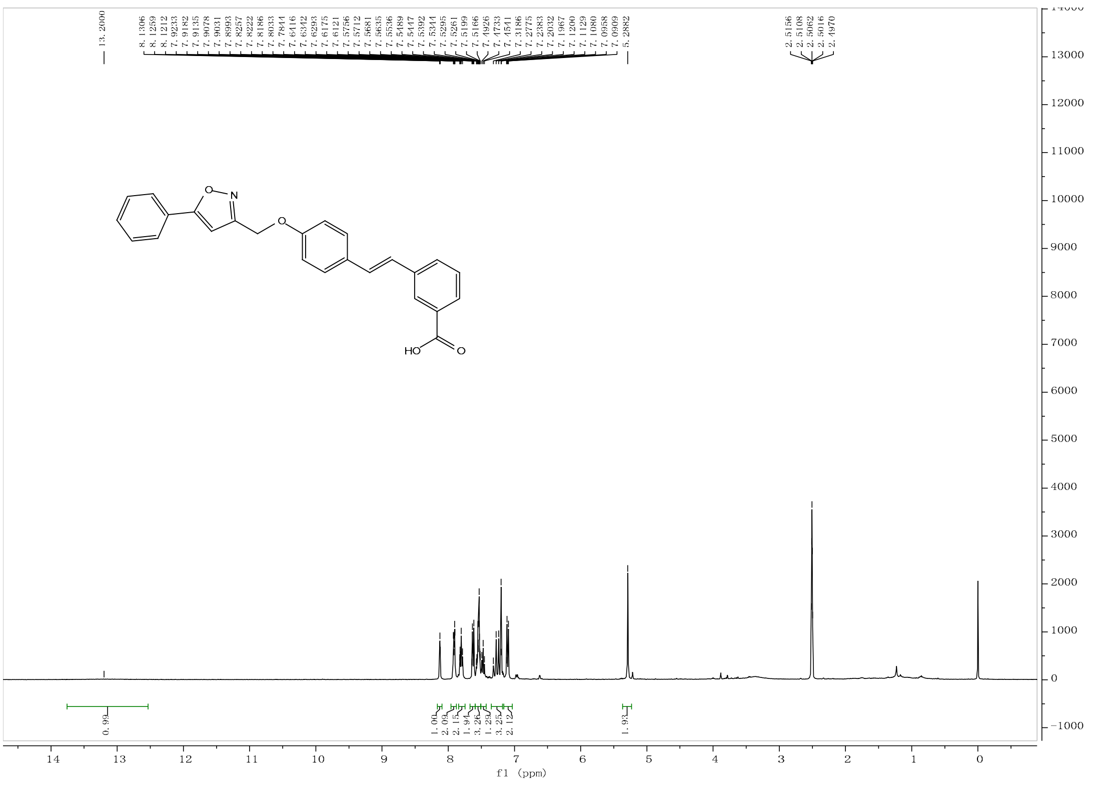

Figure S7. ^{1}H-NMR spectrum of compound **18**.

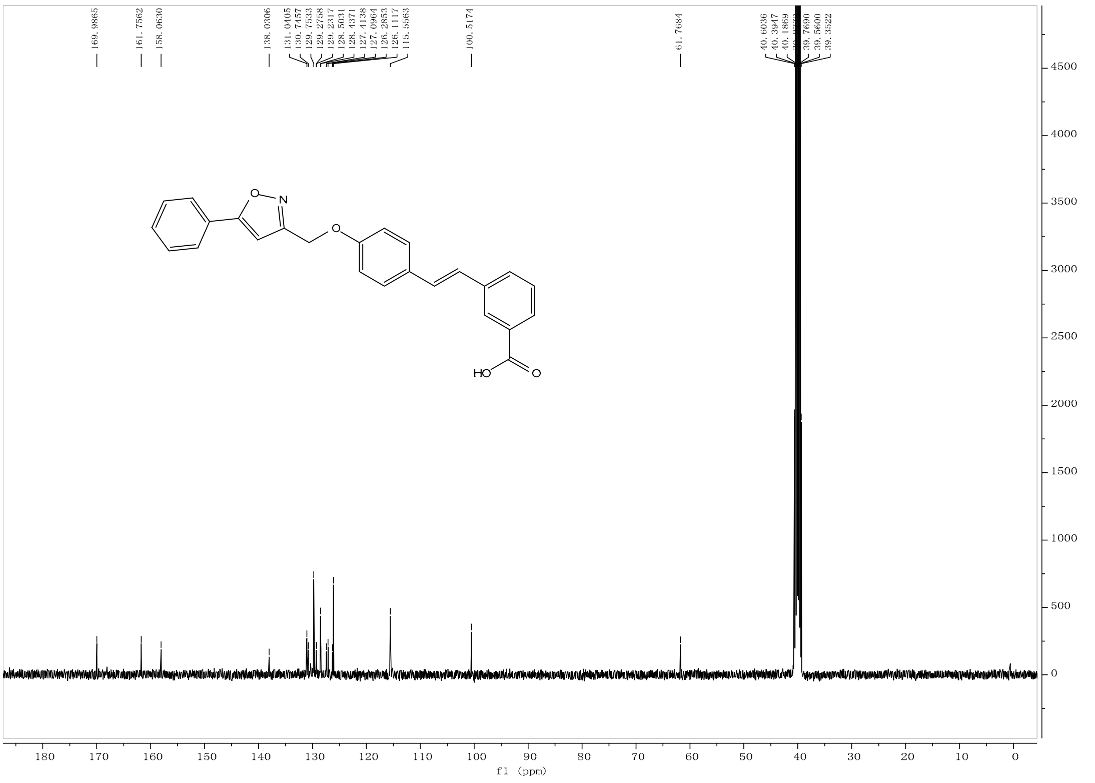

Figure S8. ^{13}C-NMR spectrum of compound **18**.

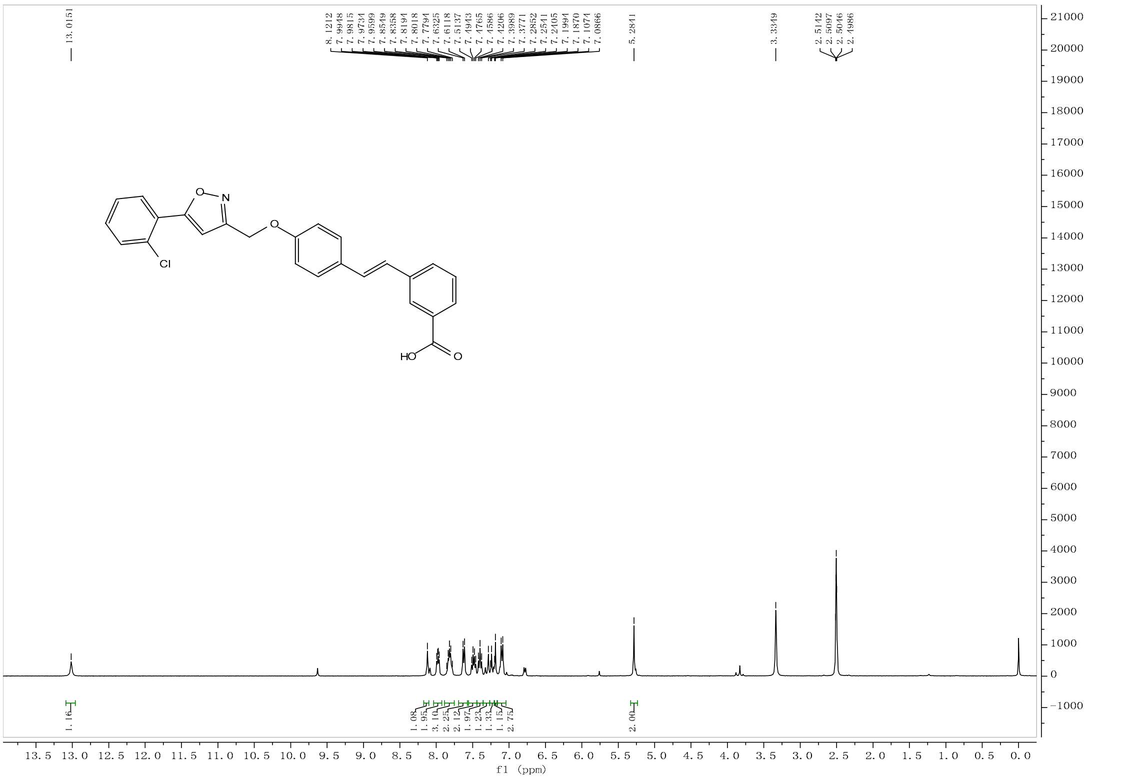

Figure S9. ^{1}H-NMR spectrum of compound **19**.

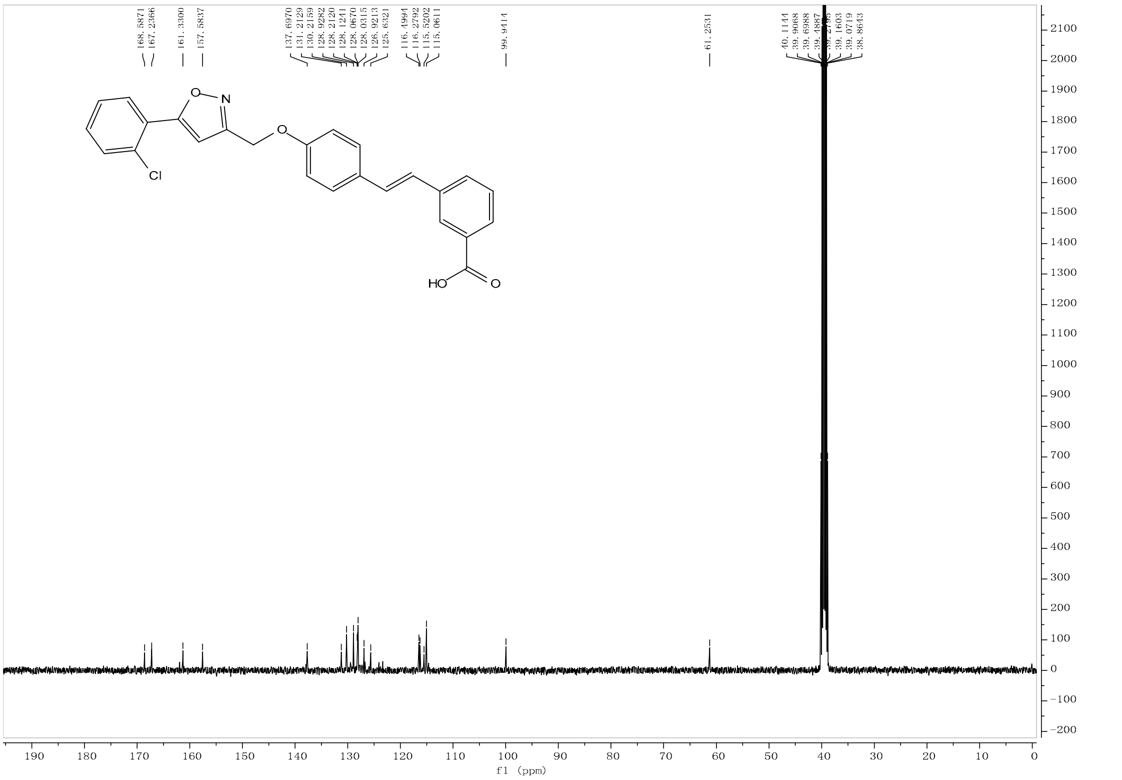

Figure S10. ^{13}C-NMR spectrum of compound **19**.

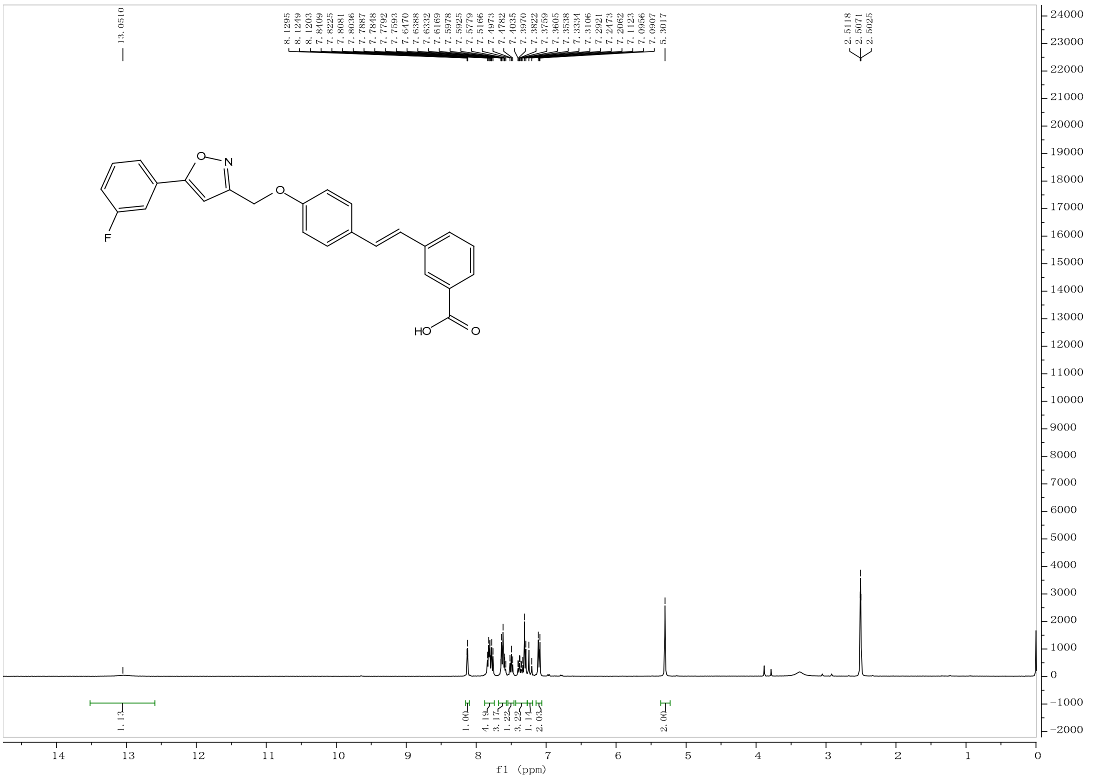

Figure S11. ^{1}H-NMR spectrum of compound **22**.

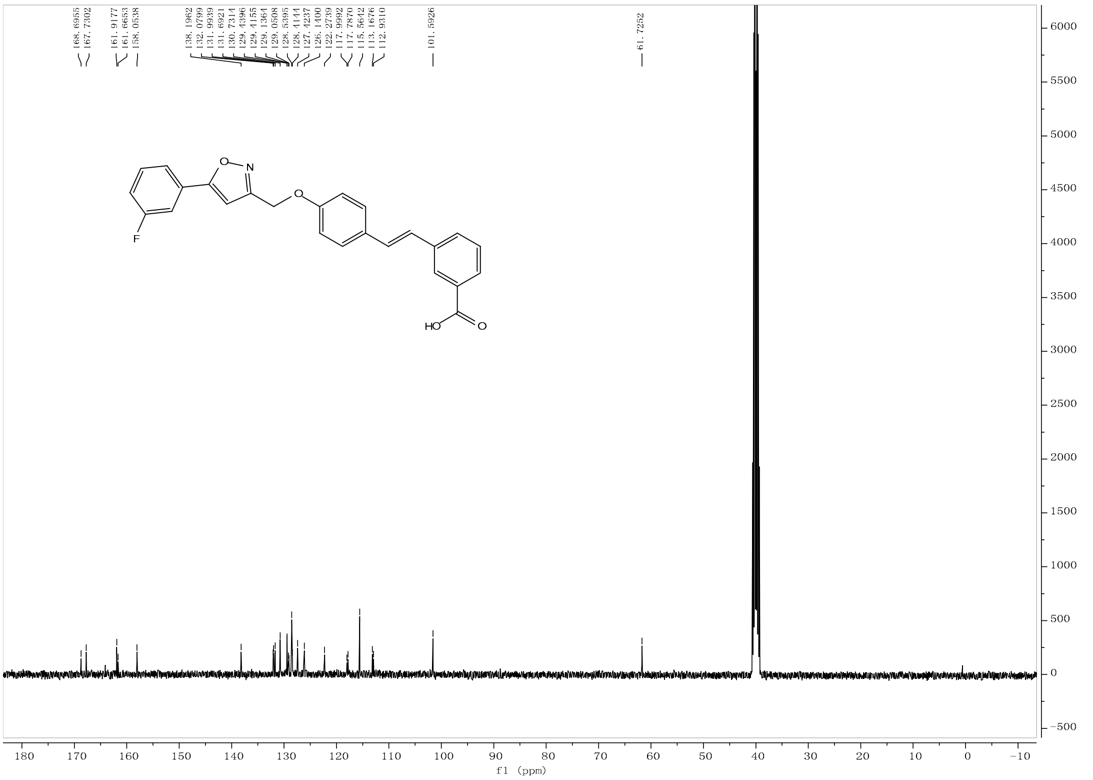

Figure S12. ^{13}C-NMR spectrum of compound **22**.

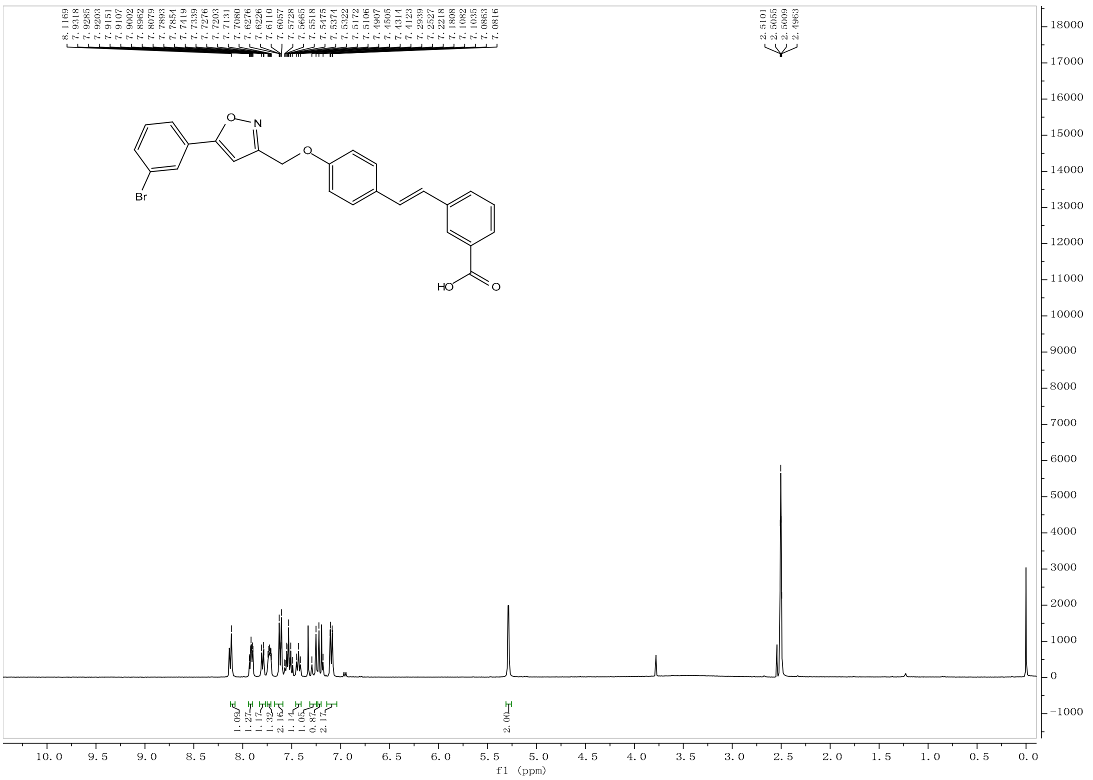

Figure S13. ^{1}H-NMR spectrum of compound **23**.

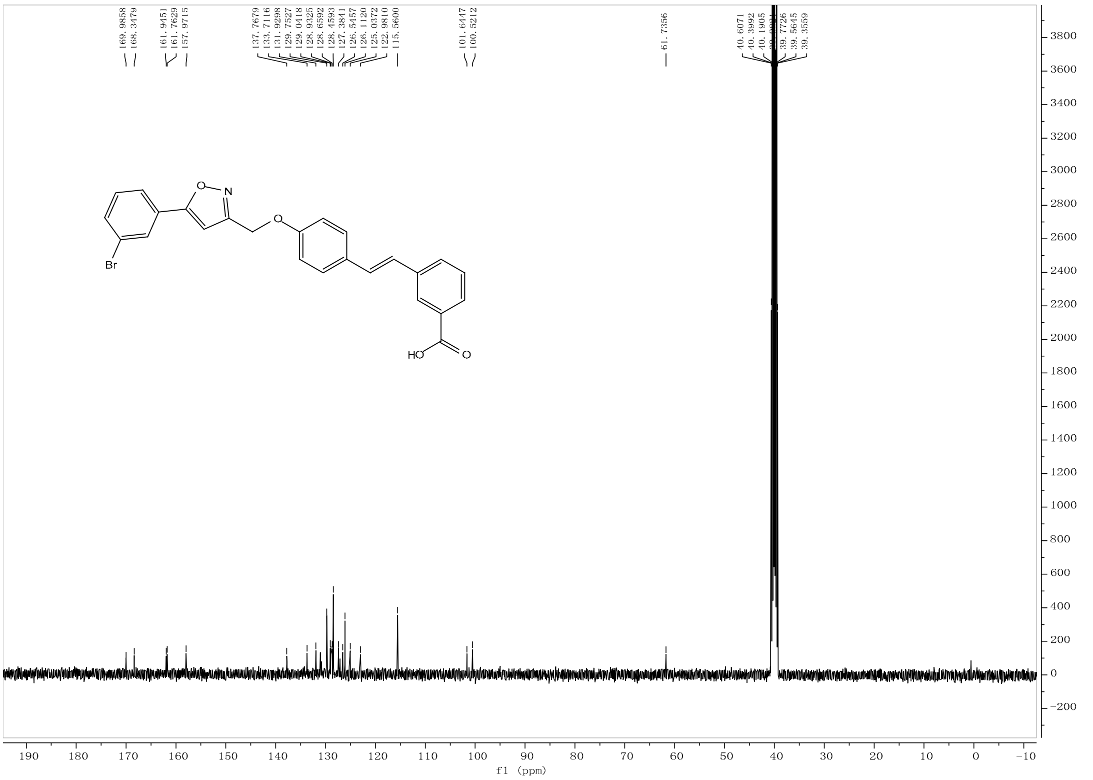

Figure S14. ^{13}C-NMR spectrum of compound **23**.

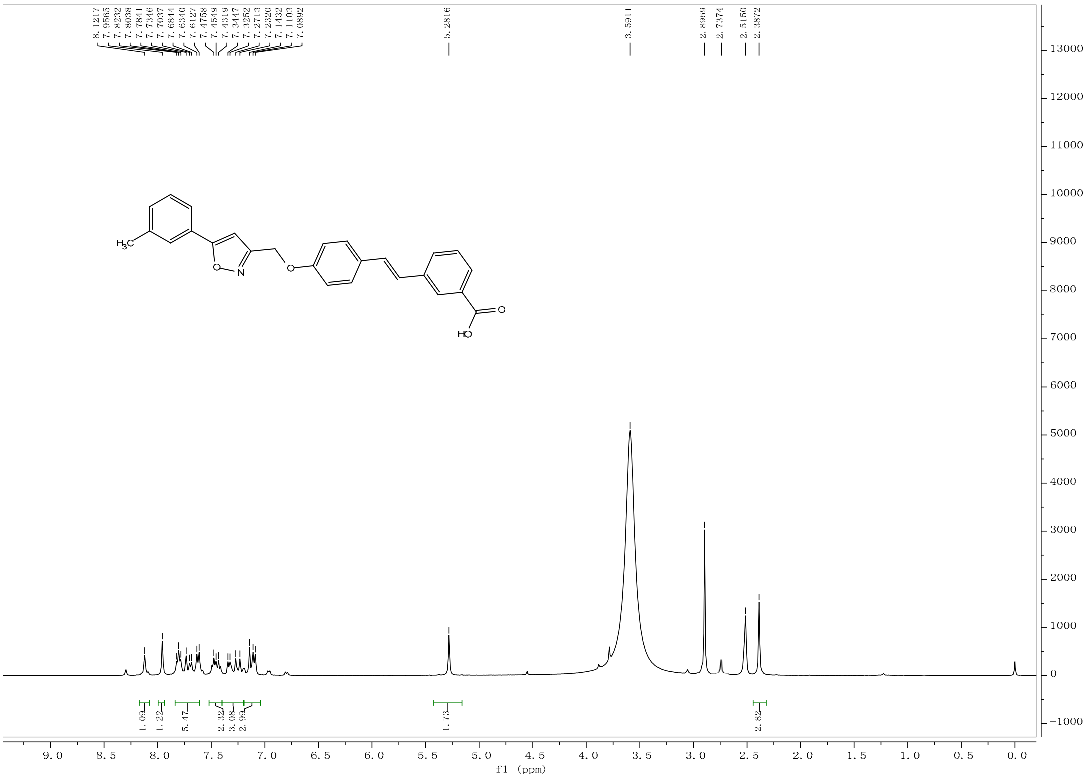

Figure S15. ^{1}H-NMR spectrum of compound **24**.

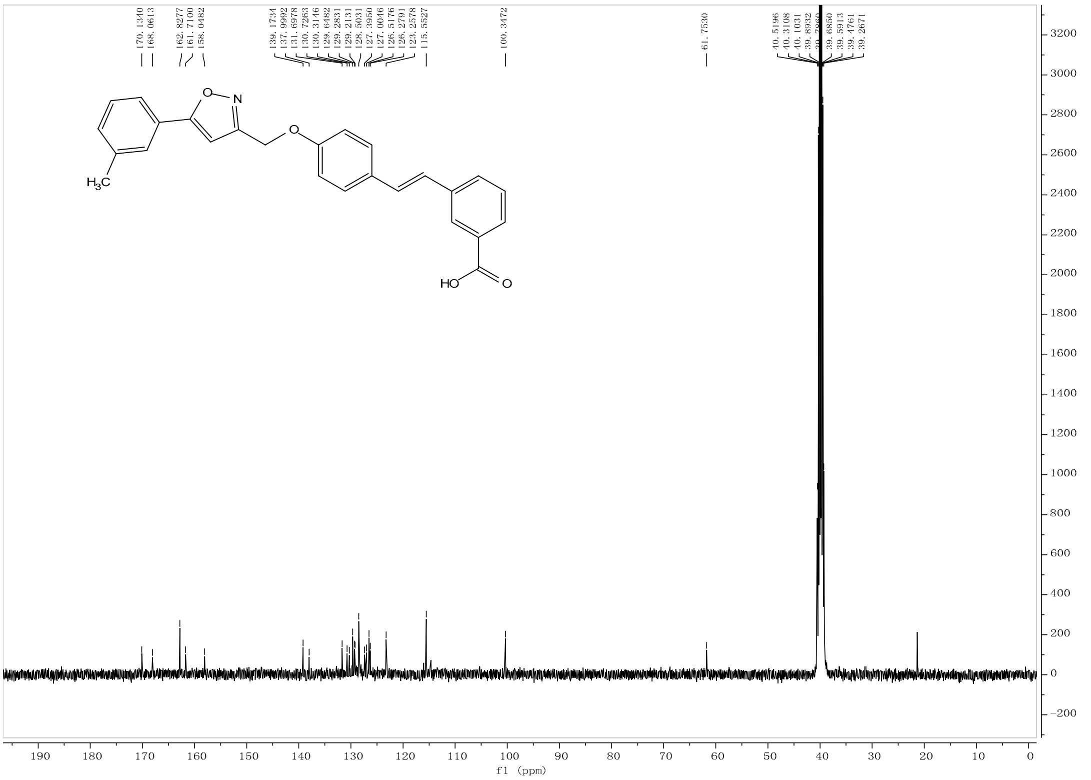

Figure S16. ^{13}C-NMR spectrum of compound **24**.

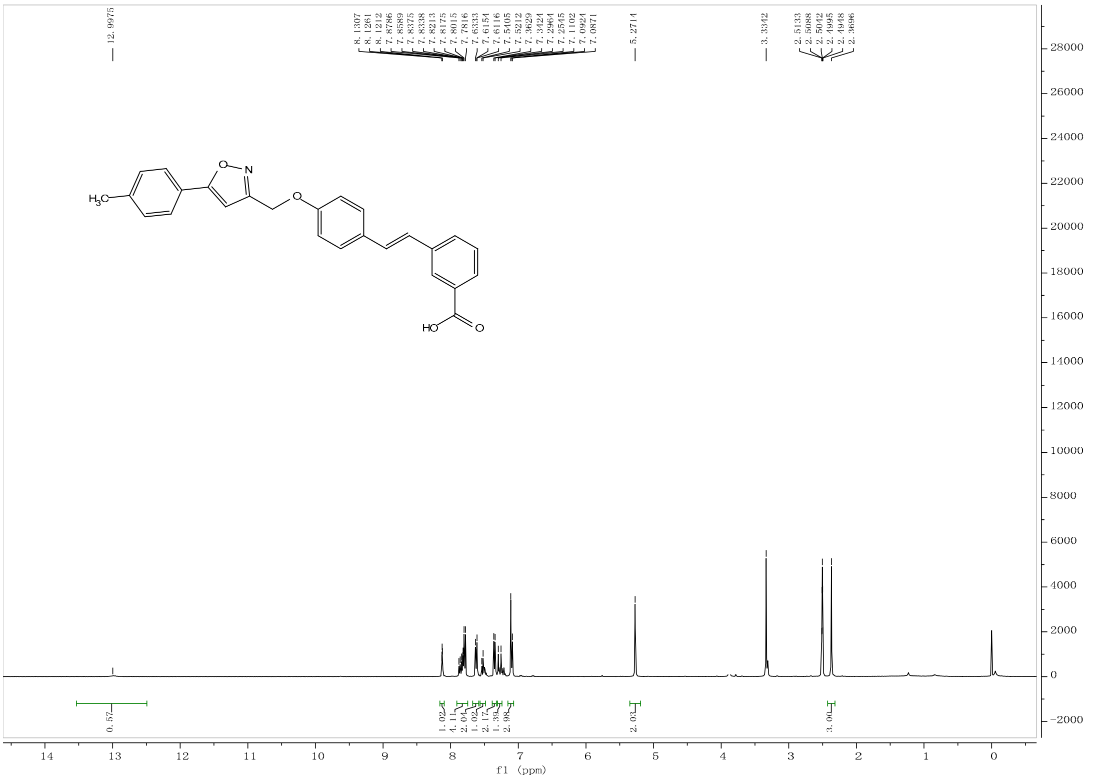

Figure S17. ^{1}H-NMR spectrum of compound **26**.

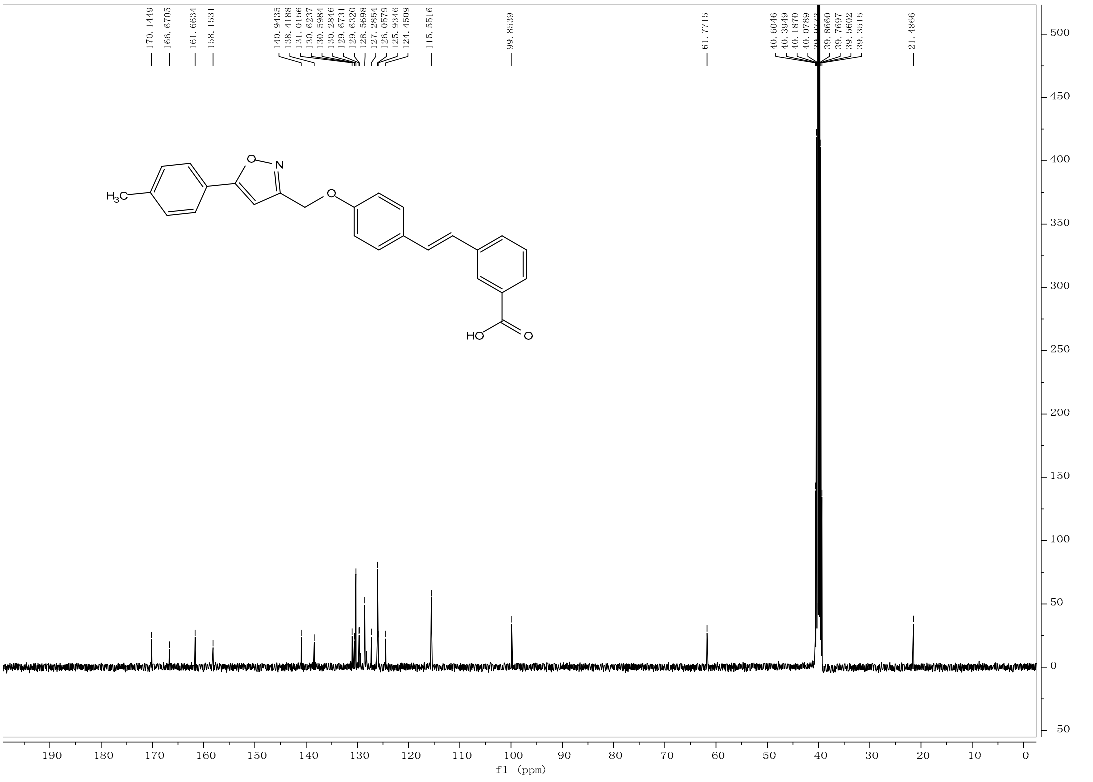

Figure S18. ^{13}C-NMR spectrum of compound **26**.

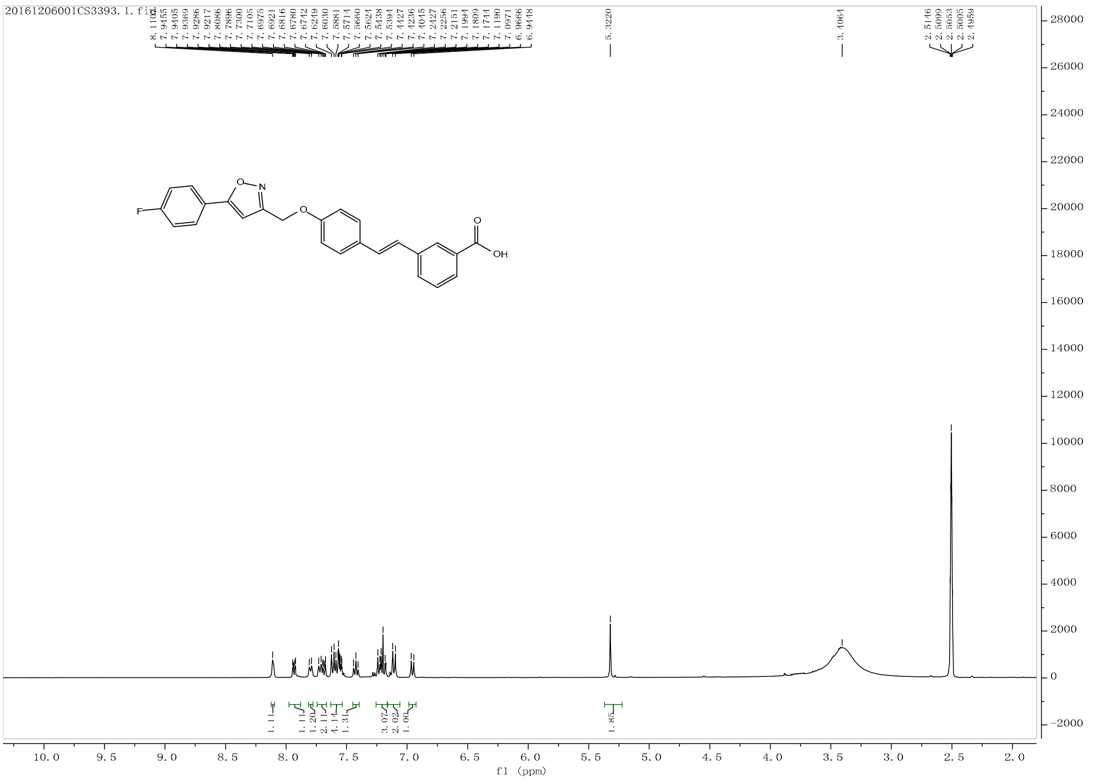

Figure S19. ^{1}H-NMR spectrum of compound **28**.

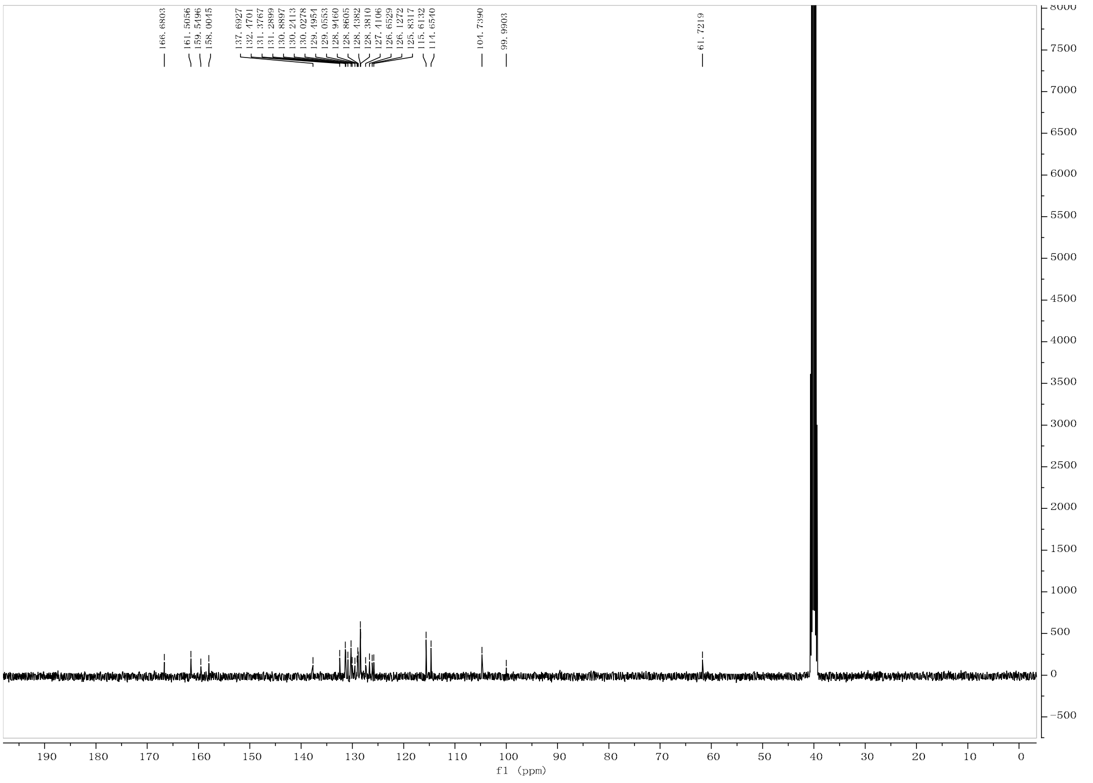

Figure S20. ^{13}C-NMR spectrum of compound **28**.

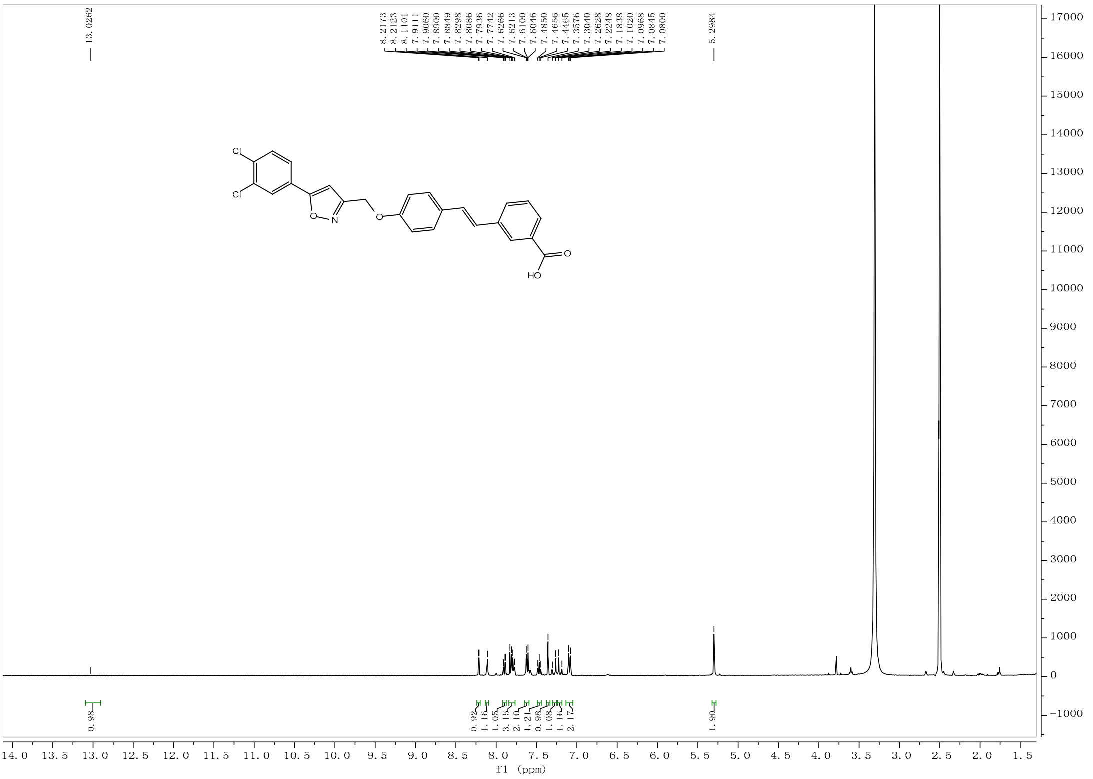

Figure S21. ^{1}H-NMR spectrum of compound **29**.

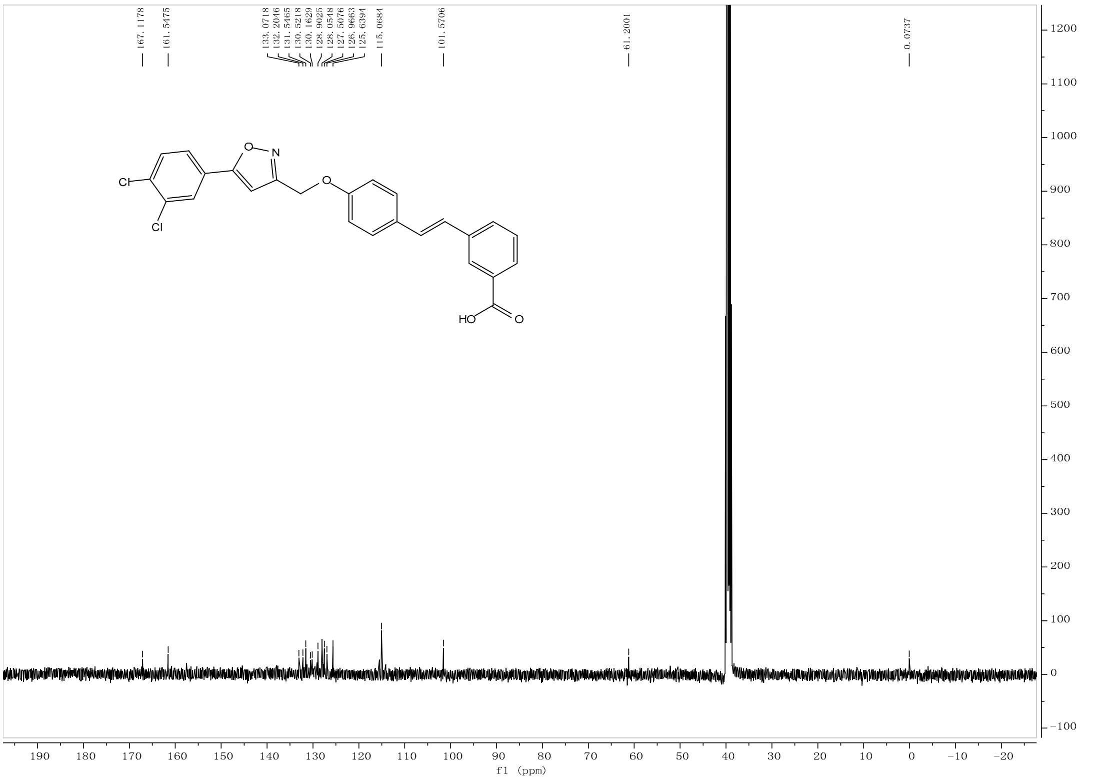

Figure S22. ^{13}C-NMR spectrum of compound **29**.

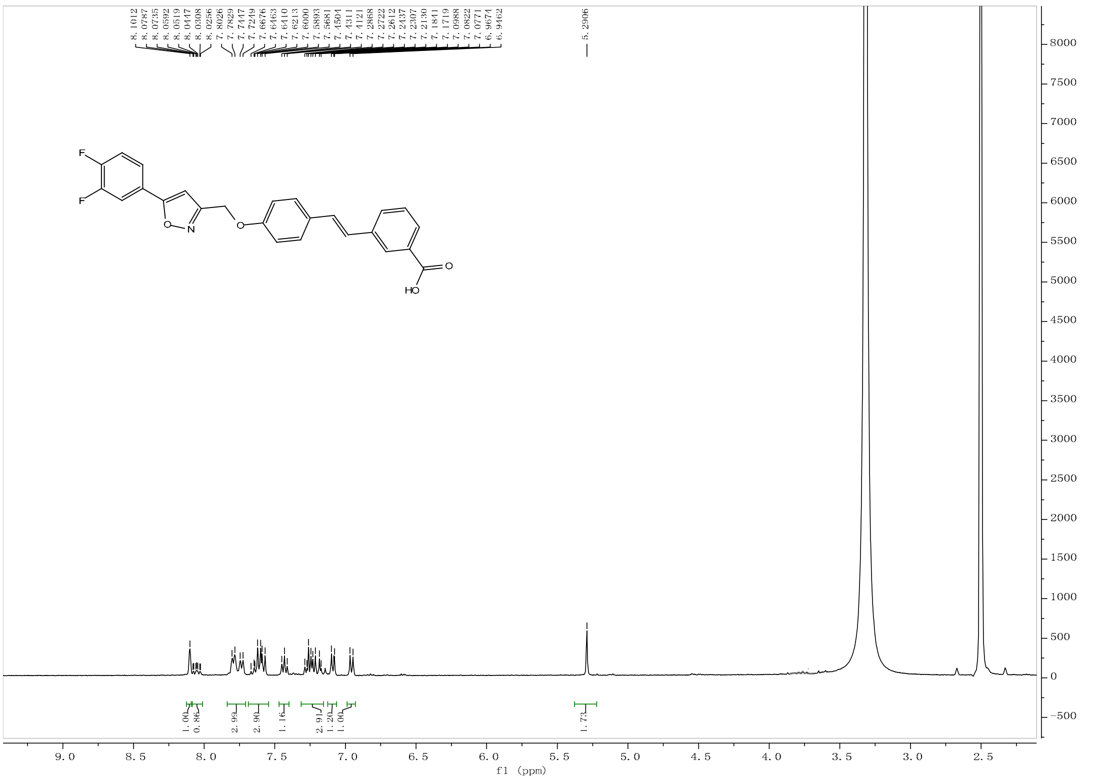

Figure S23. ^{1}H-NMR spectrum of compound **30**.

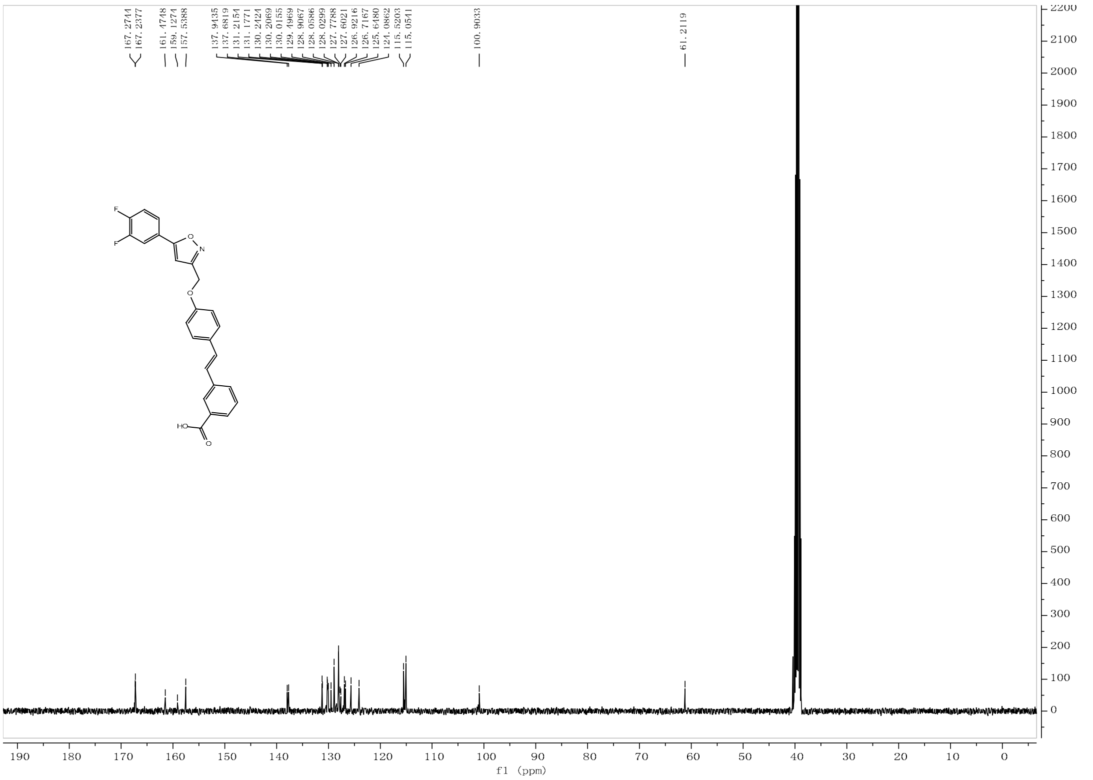

Figure S24. ^{13}C-NMR spectrum of compound **30**.
